# Exploring the potential of *Ziziphus nummularia* and luteolin-7-O-glucoside as tubulin inhibitors in cancer therapy and survival

**DOI:** 10.1038/s41598-024-57680-0

**Published:** 2024-03-26

**Authors:** Sahar Saleh Alghamdi, Sara Abdulaziz Alghashem, Rizwan Ali, Arwa Alsubait, Rasha Saad Suliman, Afrah E. Mohammed, Zeyad Alehaideb, Raghad Abdullah Alshafi, Allulu Yousef Alturki, Ishrat Rahman

**Affiliations:** 1 Pharmaceutical Sciences Department, College of Pharmacy, King Saud Bin Abdulaziz University for Health Sciences, Riyadh, Kingdom of Saudi Arabia; 2 Medical Research Core Facility and Platforms, King Abdullah International Medical Research Center (KAIMRC), Ministry of National Guard Health Affairs, Riyadh, Kingdom of Saudi Arabia; 3 King Abdulaziz Medical City, Ministry of the National Guard-Health Affairs, 11426 Riyadh, Kingdom of Saudi Arabia; 4 Department of Clinical Laboratory Sciences, College of Applied Medical Sciences, King Saud Bin Abdulaziz University for Health Sciences, Riyadh, Kingdom of Saudi Arabia; 5 Pharmacy Department, Fatima College of Health Sciences (FCHS), Abu Dhabi, United Arab Emirates; 6 Department of Biology, College of Science, Princess Nourah Bint Abdulrahman University (PNU), P.O. Box 84428, 11671 Riyadh, Kingdom of Saudi Arabia; 7 Department of Basic Dental Sciences, College of Dentistry, Princess Nourah Bint Abdulrahman University, P.O. Box 84428, 11671 Riyadh, Kingdom of Saudi Arabia

**Keywords:** *Ziziphus nummularia*, *Ziziphus spina-christi*, Anti-tubulin, Anticancer, Breast cancer, Luteolin-7-O-glucoside, Chemical biology, Computational chemistry

## Abstract

Cancer is responsible for approximately 10 million deaths worldwide, with 70% of the deaths occurring in low- and middle-income countries; as such safer and more effective anti-cancer drugs are required. Therefore, the potential benefits of *Ziziphus nummularia* and *Ziziphus spina-christi* as sources of anti-cancer agents were investigated. *Z. nummularia* and *Z. spina-christi* extracts were prepared using chloroform, ethanol, ethyl acetate, and water. The extracts’ anti-cancer properties were determined using the MTT Cell Viability Assay in four cancer cell lines: breast (KAIMRC2 and MDA-MB-231), colorectal (HCT8), and liver (HepG2). The ApoTox-Glo Triplex Assay and high-content imaging (HCI)-Apoptosis Assay were used to assess KAIMRC2 and HCT8 cells further. In addition, KAIMRC2 cells were tested for microtubule staining, and AKT/mTOR protein expression was determined by western blot analysis. Liquid chromatography-mass spectrometry (LC–MS) was performed to identify the secondary metabolites in the ethanol and ethyl acetate extracts, followed by in silico techniques to predict molecular targets and interactions, safety, and pharmacokinetic profile for identified metabolites. Out of the eight extracts, the ethanolic extract of *Z. nummularia*, exhibited the most potent activity against KAIMRC2 cells with an IC_50_ value of 29.2 μg/ml. Cancer cell treatment with the ethanolic extract of *Z. nummularia* resulted in a dose-dependent decrease in cell viability with increased apoptosis and cytotoxic effects. Microtubule staining showed a disrupted microtubular network. The ethanolic extract treatment of KAIMRC2 cells led to upregulated expression of pAKT and pmTOR. In silico studies predicted luteolin-7-O-glucoside to be a ligand for tubulin with the highest docking score (− 7.686) and similar binding interactions relative to the native ligand. Further computational analysis of the metabolites showed acceptable pharmacokinetic and safety profiles, although ethanolic extract metabolites were predicted to have cardiotoxic effects. Ethanolic extraction is optimal for solubilizing active anticancer metabolites from *Z. nummularia*, which may act by causing M-phase arrest via inhibition of tubulin polymerization. Luteolin-7-O-glucoside is the lead candidate for further research and development as an anti-cancer agent. In addition, this study suggests that herbal treatment could switch on mechanisms of adaptation and survival in cancer cells.

## Introduction

In 2020, cancer was responsible for almost 10 million deaths worldwide, with nearly 70% of cancer-related deaths occurring in low- and middle-income countries^1,2^. Cancer is defined as abnormal cell growth characterized by local tissue invasion and hyper-progression. A cancerous tumor can develop from a single abnormally proliferating cell that undergoes several alterations^3^. The process by which a normal cell transforms into an abnormal cell is not fully understood. Therefore, there is a need to develop medications to overcome these problems^4–6^.

Phytochemicals are organic substances that naturally exist in plants and possess many biological activities^7^. Plants have a diverse array of phytochemicals, including flavonoids and alkaloids, which are thought to have anticancer effects. In addition, the core moiety of any specific compound from natural products mostly has various chemical functional groups, thus providing a compound with a wide range of biological activities^7^.

The *Rhamnaceae* family comprises approximately 58 genera^8^. *Ziziphus* is a part of the *Rhamnaceae* family, a genus composed of almost 100 species with various biological properties, such as antibacterial, antidiabetic, anti-inflammatory, and anticancer properties^9^. *Z. nummularia* is also known as the Sider part of the *Ziziphus* species and is traditionally used as an antipyretic, laxative, and antitussive^10^. A considerable amount of literature has been published on *Z. nummularia*’s anti-cancer activity against multiple cell lines, including human colon adenocarcinoma (HT-29), breast cancer (MCF-7), ovarian cancer (OVCAR-3), leukemia (K-562), human kidney carcinoma (A- 498), and pancreatic Capan-2 cancer cells, with evidence of cytotoxic activity highlighting *Z. nummularia* as a plant with broad anticancer potential^10,11^. Another member of the *Ziziphus* genus is *Ziziphus spina-christi*, also known as Christ’s thorn jujube or Sidr, which is traditionally used by many cultures to treat pain and inflammation^9^. Recently, researchers have examined the anticancer activity of *Z. spina-christi* against various cancer cell lines, including human breast adenocarcinoma MCF-7, Hela, and MDA-MB-468 tumor cell lines^12,13^. The findings demonstrated that the ethanolic fraction could restrict the growth of MCF-7 cells by promoting cell cycle arrest at the G1/S phase, evidencing the ability of *Z. spina-christi* to disrupt the cell cycle, causing apoptosis^13^.

The induction of apoptosis as a therapy in cancer treatment has gained much interest in recent decades because apoptosis plays a role in maintaining hemostatic cell growth and preventing cancer^14,15^. Accordingly, the induction of apoptosis is considered an essential therapeutic approach in cancer and *Ziziphus jujuba* has been reported to induce apoptosis by the production of mitochondrial reactive oxygen species (ROS) ^16^. Previous studies have reported using various solvents for preparing herbal extracts from which anticancer activities have been observed^17^. This highlights the importance of using optimal extraction processes, considering the solvents' physiochemical properties, and determining the ability to selectively solubilize plant metabolites with various biological activities. Several attempts have been made to investigate the effects of natural products on programmed cell death, and it has been suggested that some herbal compounds can induce apoptotic pathways (e.g., quercetin induces apoptosis by inhibiting PI3K)^18^. Apoptosis can occur under physiological and pathological conditions, where a cell activates enzymes to degrade its components (e.g., DNA and proteins)^19^. Cells undergoing apoptosis can be characterized by cell rounding, retraction of pseudocodes, decreased cellular volume, chromatin condensation, nuclear dissociation, and membrane blebbing^19,20^. Apoptosis can be triggered in multiple ways, including the intrinsic mitochondrial pathway, activation of the extrinsic pathway involving the death receptor, and a caspase-independent mechanism^19^. The intrinsic and extrinsic pathways activate caspases and induce apoptosis. Caspase activation depends on producing pro-apoptotic enzymes (e.g., BAX and BAK) and downregulation of anti-apoptotic enzymes (e.g., BCL-2, BCL-XL, and MCL-1). In the intrinsic pathway, internal stimulants (e.g., high calcium (Ca^+^) concentrations in the cytoplasm and oxidative stress) activate the apoptotic pathway^20^. Initiator procaspases 8 and 9 become active upon autocatalytic cleavage, and then dimerization is induced by components of either the extrinsic pathway (activating caspase 8) or by the apoptosome generated through the intrinsic pathway (activating caspase 9)^21,22^.

In the extrinsic pathway, apoptotic signaling begins with the binding of a ligand (e.g., tumor necrosis factor and TNF-related apoptosis-inducing ligand) to the extracellular part of the death receptor (e.g., type 1 TNF receptor). Once the ligand is bound to the outer part of the death receptor, the intracellular part binds to an adaptor protein (e.g., Fas-associated protein death domain and TNF receptor-associated death domain). An adapter protein-binding site is formed, and the complete ligand-receptor-adapter protein complex is called the cell death-inducing signaling complex (DISC). Subsequently, DISC begins to evolve and activates the initiator caspase 8. The executioner caspases 3, 6, and 7 are activated upon cleavage by the initiator caspases, generating the mature and functional proteolytic activity of the executioner caspases, which finally results in cellular apoptosis^19–23^.

AKT and mTOR are integral proteins that regulate cell growth, proliferation, and genome stability. AKT, also known as protein kinase B (PKB), has three isoforms (Akt1, Akt2, and Akt3) and plays crucial roles in cell size, cycle, genome stability, protein synthesis, and angiogenesis. mTOR is an AKT downstream effector that forms mTORC1 and mTORC2, controlling cellular development, metabolism, cell survival, and proliferation. Mutations in this pathway are prevalent in cancers^24–26^. Tubulin, a target for anti-cancer drugs like taxanes and vinca alkaloids, is vital for cell structural integrity, cell cycle termination, and mitotic spindle formation. Tubulin dimers, composed of α- and β-tubulin, form microtubules essential for cell shape determination, mitotic spindle formation, cell division, and axon extension. Dynamic microtubules are critical for mitotic spindle function, and any alterations can lead to mitotic arrest or apoptosis^27–30^.

The current study advances and broadens the current understanding of the anti-cancer properties of *Z. nummularia* and *Z. spina-christi*. The biological advantages of *Z. nummular*ia and *Z. spina-christi* against cancer were the main topics of our investigation, along with a deeper mechanistic understanding of their anticancer activity. This study identified the optimal extraction solvent capable of solubilizing biologically active metabolites from *Z. nummularia* and *Z. spina-christi* to harness anticancer activities from these herbs. Furthermore, tentative identification of the secondary metabolites was conducted to perform multiple in silico investigations, including molecular targets and safety predictions that highlight their use as lead compounds for further research and development in the field of cytotoxic drug discovery.

## Material and method

### Chemicals and reagents

Dulbecco’s phosphate-buffered saline (D-PBS), Dulbecco’s Modified Eagle Medium (DMEM) plus, fetal bovine serum (FBS), GlutaMax-1 (4.5 g/L d-Glucose, 25 467 mM HEPES, Pyruvate), and TrypLE™ Express were purchased from Gibco® (Waltham, MA). The NP40 Cell Lysis Buffer (Carlsbad, CA, USA) was purchased from Thermo Fisher Scientific. Ethanol was purchased from Merck (Kenil- 472 worth, NJ). chloroform was obtained from Sigma-Aldrich (St. Louis, MO). Staurosporine was obtained from Santa Cruz Biotechnology (Dallas, TX, USA). Dimethyl sulfoxide (DMSO) was purchased from Calbiochem (San Diego, CA, USA). Ethyl acetate and ethanol were purchased from Merck (Kenil- 472 worth, NJ). Chloroform was obtained from Sigma Aldrich (St. Louis, MO). Ultra-pure water was produced using a Millipore (Billerica, MA, USA) system with a resistivity reading of 18.2 MΩ cm at 25 °C. Saudi Industrial Gas (Dammam, Saudi Arabia) provided purified carbon dioxide (CO_2_) gas.

### Herb collection

*Z. spina-christi* and *Z. nummularia* leaves were wildcrafted in the Albatin dam area near Jalajil city, Sudair region, Kingdom of Saudi Arabia (coordinates:25.6834° N, 45.4592° E), and a permission was obtained from King Abdullah International Medical Research Center (KAIMRC) with permission number RC16-175 before plants were wild crafted and collected. The identification of the plants was confirmed by Botanist Dr. Mona Alwhibi (professor of Botany and Toxonomy, King Saud University). A voucher sample was deposited at King Saud University (Herbium. Department of Botany and Microbiology) with accession numbers 24,176 and 24,322. Leaves were washed with filtered water and dried under a stream of slightly warm, dry air. After drying, the leaves were finely powdered using an electric motor grinder and stored in the dark at room temperature. The plant collection and use were in accordance with all the relevant guidelines. A voucher sample was deposited at King Saud University (Herbarium, Department of Botany and Microbiology) under accession numbers Table 1.

**Table 1:** *Z. spina-christi* and *Z. nummularia* collection data.

Herb name	Family	Plant part used	Species confirmed	KSU herbarium no	Type of collection	Sample location
*Ziziphus nummularia*	*Rhamnaceae*	Leaves	Yes by Botanist	24,176	Wildcrafted	Riyadh regional area
*Ziziphus spina-christi*	*Rhamnaceae*	Leaves	Yes by Botanist	24,322	Wildcrafted	Riyadh regional area

### Herb extraction

Approximately 500 mg of each *Z. spina-christi* and *Z. nummularia* dried leaf was extracted in 10 ml of water, ethanol, ethyl acetate, and chloroform under high-power sonication using a Sonicator (Newton, CT, USA) Vibra-Cell Ultrasonic Liquid Processor (Model GEX-130 probe-sonicator) for 3 h. The sonicated extract was filtered using a Sartorius Stedim biotech (Göttingen, Germany) quantitative ashless paper filter under gravity flow, and dried in an incubator at 40 °C. The dried residue was weighed and reconstituted in 100–500 μL of DMSO. The extract was stored in a refrigerator at 6.5 °C. The extraction and yield calculation were repeated three times. The extract yield percentages and standard deviation for *Z. spina-christi* were 12.7% ± 43.133, 12.55% ± 2.081, 4.45% ± 2.12, and 7.72% ± 1.414 in water, ethanol, ethyl acetate, and chloroform, respectively. The percentage extract yield for *Z. nummularia* and standard deviation in water was 10.1% ± 34.4, in ethanol 8.26% ± 20.9, 3% ± 7.5 ethyl acetate, and 9.8% ± 28.5 in chloroform. The yield percentages were calculated using the following formula:

$${\text{yield (\% ) = }}{{\left( {{\text{weight}}\,{\text{of}}\,{\text{the}}\,{\text{extract}}\,{\text{following}}\,{\text{solvent}}\,{\text{evaporation }} \times {\text{100}}} \right)} \mathord{\left/ {\vphantom {{\left( {{\text{weight}}\,{\text{of}}\,{\text{the}}\,{\text{extract}}\,{\text{following}}\,{\text{solvent}}\,{\text{evaporation }} \times {\text{100}}} \right)} {{\text{dry}}\,{\text{weight}}\,{\text{of}}\,{\text{the}}\,{\text{extract}}}}} \right. \kern-\nulldelimiterspace} {{\text{dry}}\,{\text{weight}}\,{\text{of}}\,{\text{the}}\,{\text{extract}}}}.$$

### In vitro anticancer activity

#### Cell culture

In vitro tests to determine the effects of *Z. nummularia* and *Z. spina-christi* extracts on cell growth and proliferation were conducted against colorectal HCT8, hepatoma HepG2, breast cancer KAIMRC2, and MDA MB-231 cell lines. All cell lines were purchased from ATCC, United States, except for KAIMRC2, which was established in KAIMRC in Riyadh, Saudi Arabia, from a 34-year-old female Saudi Arabian patient with breast cancer and data of the cell line was characterized and published previously by another research group^75^. KAIMRC2 is a triple-negative breast cancer cell (TNBC) line. For each cell line, advanced DMEM with 10% FBS, 1% L-glutamine, and 1% Pen-Strep antibiotics were used. The cells were seeded at a density of 5 × 10^3^ cells/well in 96-well plates in a 100 μL growth medium. The extract treatment was performed in triplicates and the cells were incubated at 37 °C for 48 h.

### MTT assay

The viability of HCT8, HepG2, KAIMRC2, and MDA MB-231 cells was assessed using the MTT assay^31^. Mitoxantrone served as a positive control to quantify growth inhibition. The cells were cultured in 96 well plates as described above and then exposed to each solvent extract with various concentrations of each extract ranging from 0 to 500 µg/ml and incubated for 48 h. Later, cells were incubated with 5 mg/ml MTT solvent for 4 h at 37 °C. Afterward, the media was aspirated, and DMSO was added, and the plates were shaken for 45 min on an orbital shaker. The absorbance was measured at OD590 nm. Finally, the half-maximal inhibitory concentration (IC_50_) values (µg/ml) were calculated from the dose–response curves.

### ApoTox-Glo™ triplex assay

CellTiter-Glo assay (Promega, Madison, WI, USA) was used to determine the number of viable cells in the culture by counting the amount of ATP present to identify cells with an active metabolism. This assay tested the viability, cytotoxicity, and apoptosis of KAIMRC2 and HCT8 cells^32^. First, KAIMRC2 and HCT8 cells were seeded in 96-well plates (Costar, Thermo 529 Fisher Scientific) and then treated with various concentrations of either ethanolic or ethyl acetate extracts of *Z. nummularia* . Cells were also treated with positive control, mitoxantrone. The plates were incubated for 48 h at 37 °C. After treatment, ApoTox-Glo™ Triplex Assay kit was used as described by the manufacturer^33^. The luminescence was then measured at the following two-wavelength sets using an Envision plate reader (Perkin Elmer) 400ex/505em (viability) and 485ex/520em (cytotoxicity). To measure the level of apoptosis i.e., caspase 3/7 activation, the luminescence was measured using a Perkin Elmer plate reader. The data were plotted on graphs using Microsoft Excel™.

### HCI-apoptosis assay

The HCI-based Live/Dead/Apoptosis Assay is a multiplexed cell-staining assay that is used to understand the biological functions and mechanisms of drug action. KAIMRC2 and HCT8 cells were seeded into 96-well plates at a density of 20,000 cells/well. The cells were treated with increasing concentrations (34.37 μg/mL, 68.75 μg/mL, 137.5 μg/mL, and 275 μg/mL) of *Z. nummularia* in ethanol and *Z. nummularia* ethyl acetate for 48 h. Mitoxantrone treatment was used as a positive control and DMSO was used as a negative control. After treatment, the cells were stained with HOECSHT33342 (2.5 µg), Propidium Iodide (2.5 μg/mL), and YoPro-1 (2 µg/mL) for ~ 45 min at 37 °C and 5% CO_2_. Plates were scanned using the Molecular Devices ImageXpress® Microsystem, and the captured image data were examined using the MetaXpress® software (Downingtown, PA, USA). The Cell Health module of MetaXpress software was used to determine the percentage of viable and dead cells. Each experiment was performed in triplicates.

### Microtubule staining

Microtubule staining was performed according to the standard protocol for microtubule-associated proteins and tubulin (MAPs). KAIMRC2 cells were treated with *Z. nummularia* in ethanol and *Z. nummularia* in ethyl acetate for 48 h and then stained with tubulin tracker Green 51 (Cat #T34075) for 30 min and HOECHST33342 for 5 min, after which the cells were washed and the media replaced with HBSS. The images were acquired using a Zeiss laser-scanning 780 microscope and the image analysis was performed in the ZEN black version^30^.

### Western blot assay

After protein extraction from untreated and *Z. nummularia* ethanolic extract-treated KAIMRC2 cells, equal amounts of protein (around 15 µg per well) were separated by 10% SDS-PAGE gel electrophoresis and transferred to a polyvinylidene fluoride (PVDF) membrane. Nonspecific binding sites were blocked with phosphate-buffered saline containing 0.1% Tween 20 and 5% bovine serum albumin (BSA) for 1 h at room temperature. The membrane was then incubated with specific primary antibodies against p-mTOR (Ser2448, Invitrogen), Invitrogen Anti-mTOR Monoclonal (215Q18), Phospho-Akt (Ser473) (D9E) Cell signaling), and AKT (pan, 40D4, Cell signaling). Sample loading was determined by probing with a mouse monoclonal antibody against the β-actin loading control (Thermo Fisher Scientific; 1:1000). Signals were detected using a ChemiDoc MP system (Bio-Rad, Hercules, CA, USA) and analyzed using the Image Lab software.

### LC-QTOF-MS

An Agilent 1260 Infinity HPLC system (Agilent, Germany) and an Agilent 6530 Quadrupole Time of Flight (Agilent, Singapore) were used to analyze the extracts. Separation was performed using Agilent Extend-C18 column (2.1 mm × 50 mm, 1.8 μm) with the following elution gradient; 0–1 min, 5% B; 1–11 min, 5–100% B; 11–13 min, 95% B; 13–15 min, 5% B; 15–16 min, 5% B using mobile phase A (0.1% HCOOH in water) and mobile phase B (0.1% HCOOH in Methanol). The flow rate was set to 120 µL/min, and the injection volume was 10 µL. Positive-mode MS1 was acquired using a mass range of 100–600 m/z. The following settings were used for the mass spectrometer: sheath gas temperature, 350 °C; sheath gas flow 11, gas temperature, 300 °C; gas flow, 8 µL/min; and nebulizer, 35 psig. The Agilent Mass Hunter qualitative analysis software created the MS1 data.

### Statistical analysis

The log-inhibition variable slope (three or four parameters) model was used to calculate the inhibition and effective doses using GraphPad Prism (San Diego, CA) software version 9.5.1. The Student's t-test was used to determine whether the treatment was statistically different from the untreated control, and a significant difference was considered when the p-value was less than 0.05. The results are reported as the mean ± SD.

### In silico methods

#### Predictions of anti-cancer activity

The two-dimensional chemical structures of the identified metabolites that were identified using LC-QTOF-MS were used to predict anti-cancer activity using the Prediction of Activity Spectra for Substances (PASS) webserver (http://way2drug.com/PassOnline/)^34^. Regarding the experimentally active compounds, the prediction outcomes gave an activity score (P_a_), indicating that the metabolite was active if the score was greater than 0.7, moderately active if it was within the range of 0.5–0.7, or inactive if it was lower than 0.5.

#### Predictions of molecular target

The molecular targets of the identified metabolites were predicted using Molinspiration Chemoinformatics tools (https://www.molinspiration.com/cgi-bin/properties), which provide an estimated bioactivity score for metabolites against a variety of biological targets including G protein-coupled receptors (GPCR), ion channels, nuclear receptors, kinases, proteases, and enzymes. The bioactivity score is used to categorize compounds as either active (predicted score equal to or greater than 0.00), moderately active (predicted score between − 0.50 and 0.00), or inert (predicted score less than − 0.50) against targets^35^.

### Molecular docking study

The crystal structure of tubulin was downloaded from the Protein Data Bank (PDB, https://www.rcsb.org/) database with crystal structure ID 4O2B. The molecular docking study was conducted using the Maestro Schrödinger Release 2022-3 software^36^. The protein was prepared using a protein preparation tool, followed by ligand preparation (metabolites were identified experimentally using LC-QTOF-MS) using LigPrep to minimize and generate several conformations. One-step docking validation was performed to validate the docking protocol, and receptor grid generation was obtained for the active site. Docking was conducted using Glide, and post-docking analysis was performed for the protein–ligand complexes.

### Pharmacokinetic parameters evaluation

Using the Swiss ADME webserver (http://www.swissadme.ch/), several pharmacokinetic parameters were predicted for the identified metabolites using the Swiss ADME webserver to evaluate and approximate the drug-like properties of oral bioavailability^37^. These variables include the central nervous system (CNS) distribution, Lipinski's rule of five (ROF), and predicted inhibition of important cytochrome P450 enzymes.

### Toxicity assessment

The toxicity of chemicals (ProTox-II) webserver (https://tox-new.charite.de/protox_II/)was used to predict and estimate several toxicity endpoints for the identified metabolites, including hepatotoxicity, cytotoxicity, carcinogenicity, mutagenicity, and immunotoxicity^38^.

### Prediction of cardiac toxicity

Using the pred-hERG 4.2 webserver (http://predherg.labmol.com.br/), the identified metabolites were computationally tested for their potential to block hERG K^+^ channels, which are linked to cardiac toxicity. The output is a probability map showing the participation of atoms in the blockage of hERG K^+^ channels, which categorizes the compounds as non-blockers, weak/moderate blockers, or strong blockers. Green represents blockage, red represents non-blockage, and grey represents no blockage at all^39^.

### Predictions of endocrine disruptome

Using the online, publicly available web server (http://endocrinedisruptome.ki.si.), the metabolites were computationally screened and evaluated for binding to 14 nuclear receptors, including the androgen receptor (AR); estrogen receptors (ER) α and β; glucocorticoid receptor(GR); liver X receptors (LXR) α and β; mineralocorticoid receptor (MR); peroxisome proliferator-activated receptors (PPAR) α, β/δ, and γ; progesterone receptor (PR); retinoid X receptor (RXR) α; and thyroid receptors (TR) α and β. The 14 receptors were docked into by the web server using the docking interface for the Target Systems (DoTS) approach^40^.

### Institutional review board statement

The research received approval from the Institutional Review Board (IRB) of KAIMRC, and the study protocols were reviewed and approved by the KAIMRC Committee.

## Results

### MTT cytotoxicity assay of *Z. nummularia* and *Z. spina-christi* extracts on different cell lines

**Figure 1:**
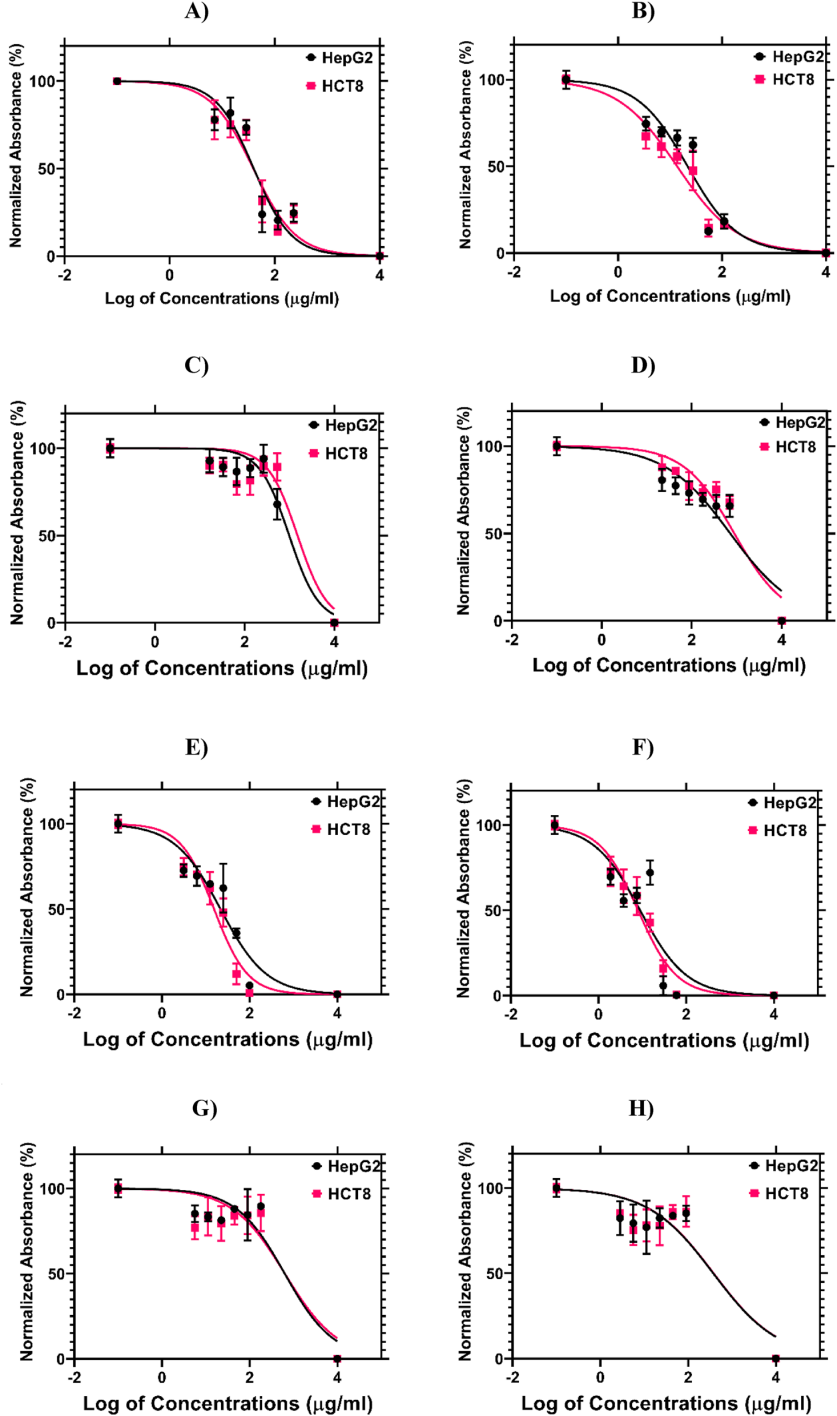
Dose–response curve for the half-maximal inhibitory concentration IC_50_ (μg/ml) of all extracts against HCT8 and HepG2 cell lines. (**A**) *Z. spina-christi* ethanol extract (**B**) *Z. nummularia* ethanol extract. (**C**) *Z. spina-christi* chloroform extract (**D**) *Z. nummularia* chloroform extract (**E**) *Z. spina-christi* ethyl acetate extract (**F**) *Z. nummularia* ethyl acetate extract. (**G**) *Z. spina-christi* water extract. (**H**) *Z. nummularia* water extract.

The anticancer activity of Z*. nummularia* and *Z. spina-christi* in chloroform, ethanol, ethyl acetate, and water extracts were examined against liver cancer cell line (HepG2), breast cancer cell lines (KAIMRC2 and MDA-MB-231), and colorectal cancer cell lines (HCT8). Figure 1 shows the dose–response curve for the half-maximal inhibitory concentration IC_50_ (μg/ml) of all the extracts against HCT8 and HepG2 cell lines using the MTT assay. Mitoxantrone was used as a positive control to evaluate growth inhibition.

The highest activity was observed in the ethanol and ethyl acetate extracts of *Z. nummularia.* The IC_50_ values for *Z. nummularia* ethanol extract were 137.0 μg/ml against HCT8 and 217 μg/ml against HepG2. While the IC_50_ values of *Z. nummularia* in ethyl acetate were 79.94 μg/mL against HCT8 and 88.99 μg/mL against HepG2 as summarized in Table 2.

**Table 2:** Z. *nummularia* and *Z. spina-christi* ethanolic, chloroform, ethyl acetate, and water extracts IC_50_ values from HCT8 and HepG2 cell lines.

Herb name and solvent	HCT8 IC_50_ (μg/ml)	HepG2 IC_50_ (μg/ml)
*Z. spina-christi* in Ethanol	391.8	399.2
*Z. nummularia* in Ethanol	137	217.6
*Z. spina-christi* in Chloroform	15,080	9838
*Z. nummularia* in Chloroform	8815	7160
*Z. spina-christi* in Ethyl acetate	154.6	217.1
*Z*.* nummularia* in Ethyl acetate	79.94	88.99
*Z. spina-christi* in Water	6349	6313
*Z*.* nummularia* in Water	3290	3740

**Figure 2:**
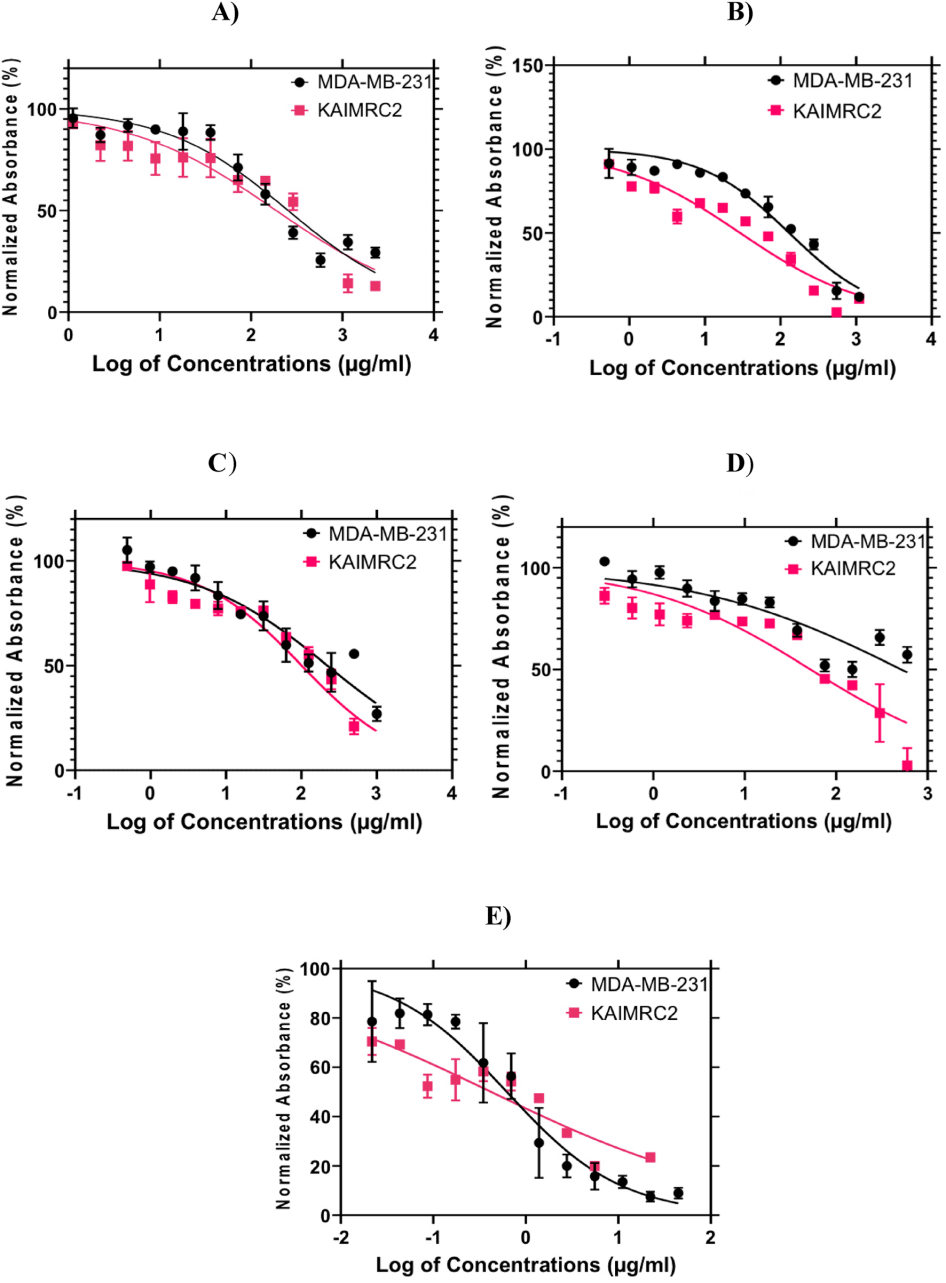
Cytotoxic effects of Z.* nummularia* and *Z. spina-christi* extracts on KAIMRC2 and MDA-MB 231 breast cancer cell lines. (**A**) Ethanol extract of* Z. spina-christi*. (**B**) *Z. nummularia* ethanolic extract. (**C**) *Z. spina-christi* ethyl acetate extract. (**D**) *Z. nummularia* ethyl acetate extract. (**E**) Mitoxantrone as a positive control.

Chloroform and water extracts had extremely high IC_50_ values. Consequently, the water and chloroform extracts were not subjected to additional testing in KAIMRC2 and MDA-MB-231 cell lines. Ethanol and ethyl acetate were used for further testing against KAIMRC2 and MDA-MB-231 cells as shown in Fig. 2**.** The IC_50_ values for *Z. nummularia* ethanol extract were 29.20 μg/ml KAIMRC2 and 130.0 μg/ml MDA-MB-231. While the IC_50_ values of* Z. nummularia* in ethyl acetate were 52.34 μg/mL against KAIMRC2, and 514.5 μg/mL against MDA-MB-231as shown in Table 3.

**Table 3:** The IC_50_ values of *Z*.* nummularia* and *Z. spina-christi* in ethanol and ethyl acetate extracts using KAIMRC2 and MDA-MB-231 cell lines.

Herb name and solvent	KAIMRC2 IC_50_ (μg/ml)	MDA-MB-231 IC_50_ (μg/ml)
*Z. spina-christi* in Ethanol	188.1	254.3
*Z. nummularia* in Ethanol	29.20	130
*Z. spina-christi* in Ethyl acetate	100.7	220.5
*Z. nummularia* in Ethyl acetate	52.34	514.5

### *Z. nummularia* against KAIMRC2 and HCT8 cancer cells lines using the ApoTox-Glo™ triplex assay.

**Figure 3:**
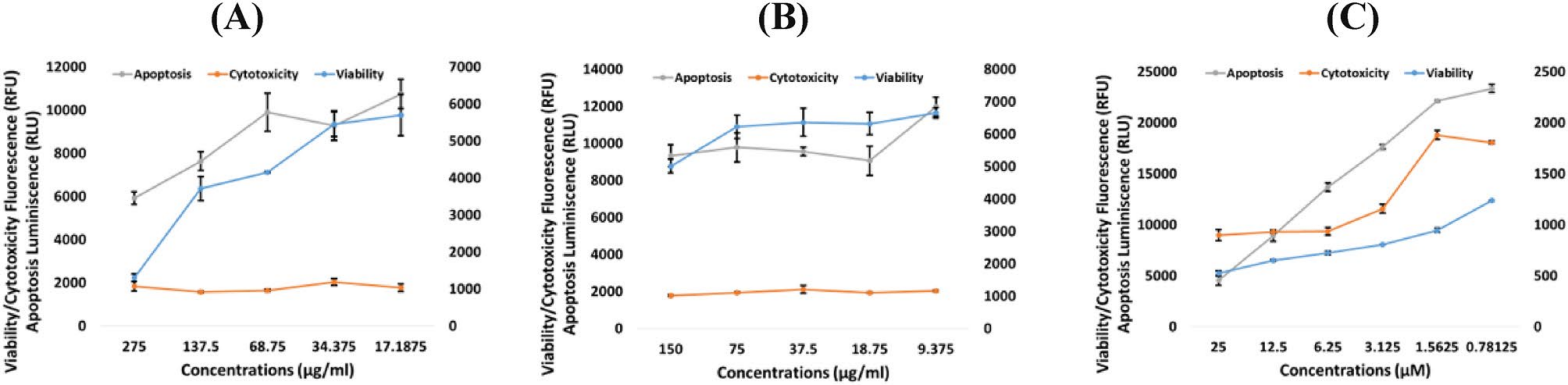
ApoTox-Glo™ Triplex Assay of *Z. nummularia* on KAIMRC2 cell line (**A**) *Z. nummularia* ethanol extract, (**B**) *Z. nummularia* ethyl acetate extract, and (**C**) Mitoxantrone.

**Figure 4:**
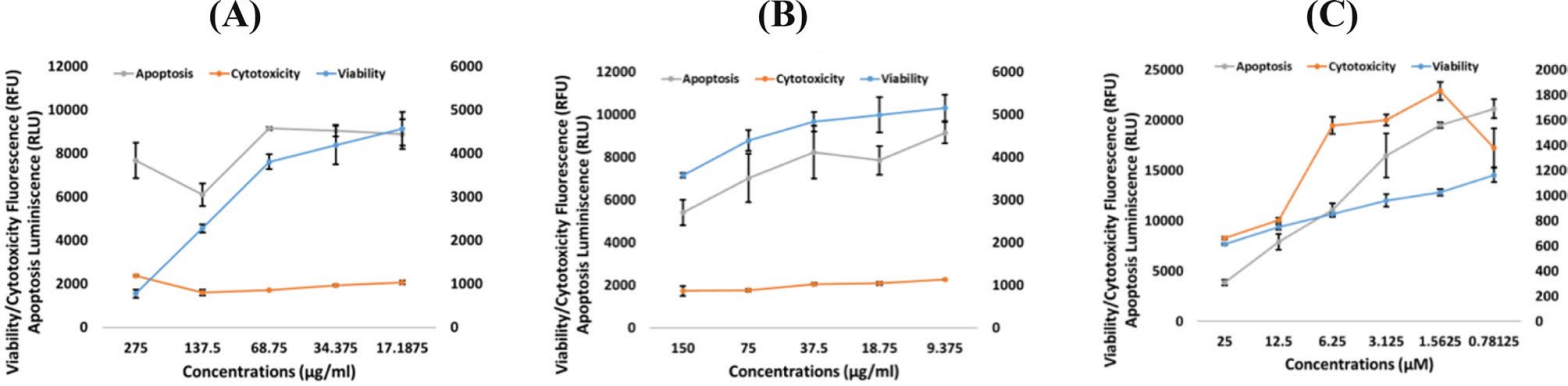
ApoTox-Glo™ Triplex Assay of *Z. nummularia* on HCT8 cell line (**A**) *Z. nummularia* ethanol extract, (**B**) *Z. nummularia* ethyl acetate extract, and (**C**) Mitoxantrone.

The ethanolic and ethyl acetate extracts of *Z. nummularia* were chosen for additional testing because, in the previous experiments, they demonstrated the highest cytotoxic activity against KAIMRC2 and HCT8 cell lines. The ApoTox-Glo Triplex Assay was used to confirm the MTT assay results. HCT8 and KAIMRC2 cell lines were treated with the ethanolic and ethyl acetate extracts of* Z. nummularia*, and mitoxantrone was used as a positive control. As illustrated in Figs. 3 and 4, the cell viability decreased in a dose-dependent manner.

### Apoptotic efficacy of *Z. nummularia* extracts on KAIMRC2 and HCT8 cancer cell lines using the HCI-apoptosis assay

**Figure 5:**
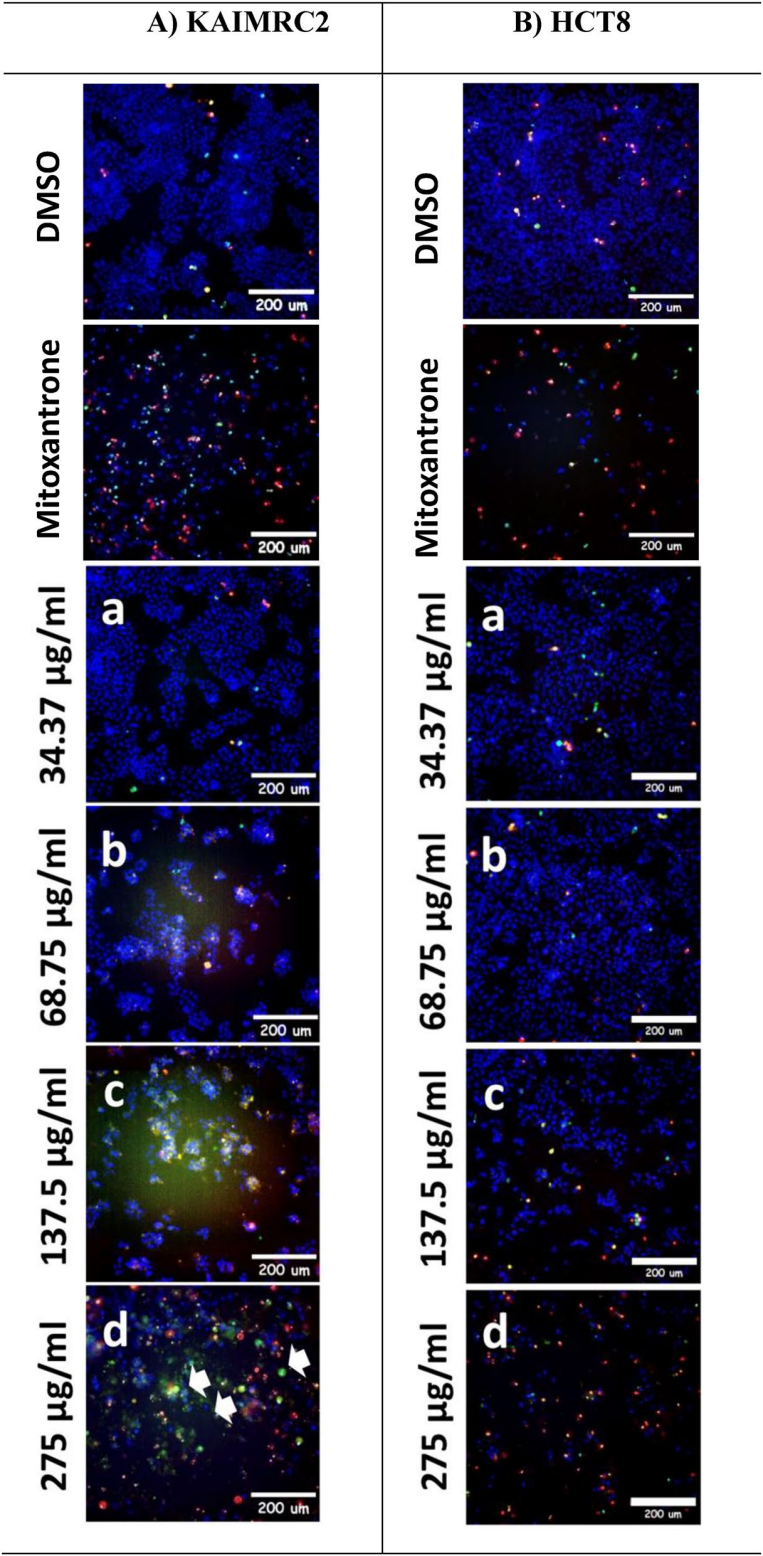
High Content Imaging (HCI) of KAIMRC2 cells (**A**) and HCT8 cells (**B**) treated with various concentrations of *Z. nummularia* ethanolic extract for 48 h. Cells were stained with HOECSHT 33,342 staining the nucleus in blue color, Propidium Iodide (PI) staining dead cells in red color, and YoPro-1 in green color showing apoptotic cells. Mitoxantrone treatment was used as a positive control and DMSO was used as a negative control. The images shown above are representative of three independent images.

**Figure 6:**
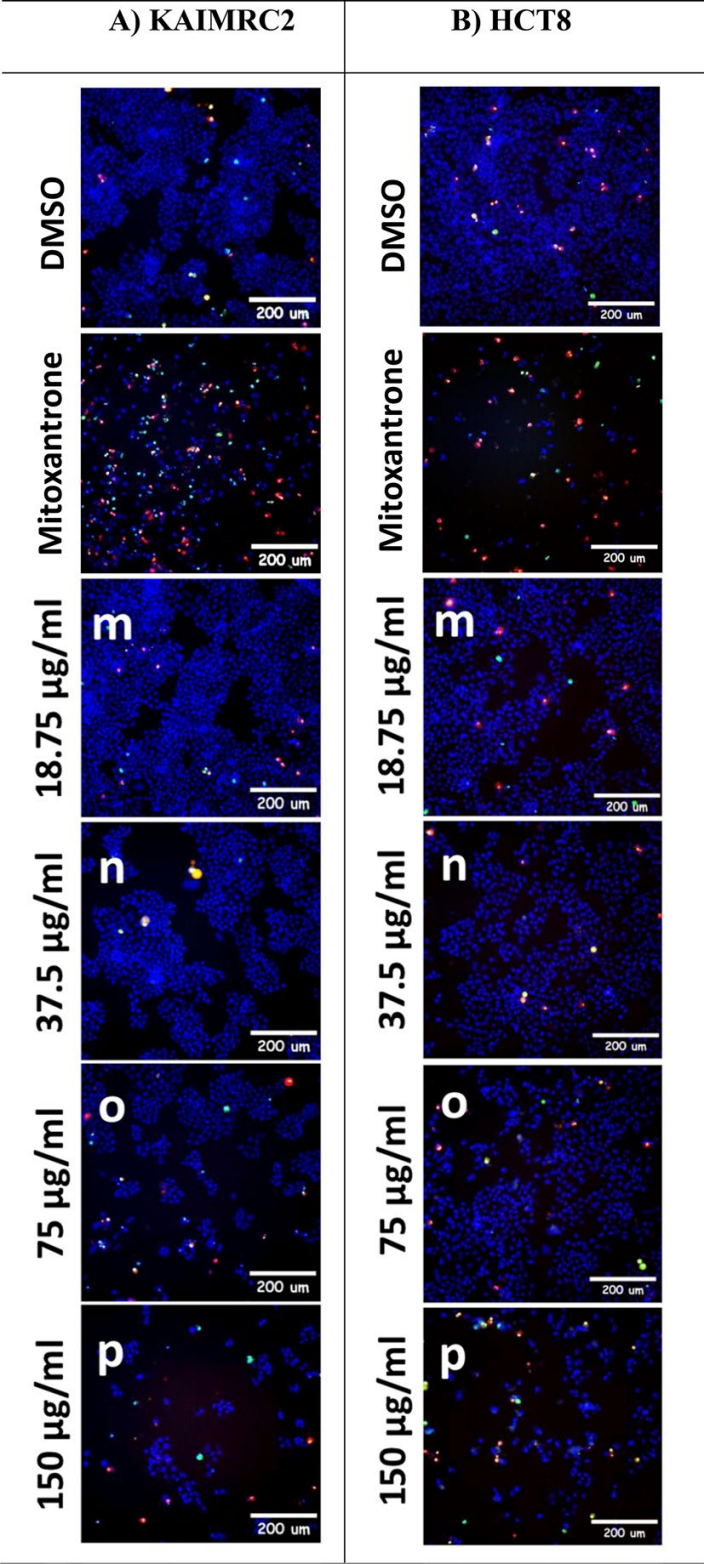
High Content Imaging (HCI) of KAIMRC2 cells (**A**) and HCT8 cells (**B**) treated with various concentrations of *Z. nummularia* ethyl acetate extract for 48 h. Cells were stained with HOECSHT 33,342 staining the nucleus in blue color, Propidium Iodide (PI) staining dead cells in red color, and YoPro-1 in green color showing apoptotic cells. Mitoxantrone treatment was used as a positive control and DMSO was used as a negative control. The images shown above are representative of three independent images.

The apoptotic effects of the ethanolic and ethyl acetate extracts of *Z. nummularia* were determined in HCT8 and KAIMRC2 cells using the HCI-Apoptosis assay. Both cells were exposed to various concentrations of each extract. Both extracts induced a dose-dependent cytotoxic effect on HCT8 and KAIMRC2 cancer cells, although the ethanolic extract of *Z. nummularia* appeared significantly more potent than the ethyl acetate extract as shown in Figs. 5 and 6. Furthermore, *Z. nummularia* ethanolic extract displayed a dose-dependent effect of inducing apoptosis in both cancer cell lines, as shown in Fig. 5**.**

### *Z. nummularia* destabilizes tubulin network in KAIMRC2 cell lines

**Figure 7:**
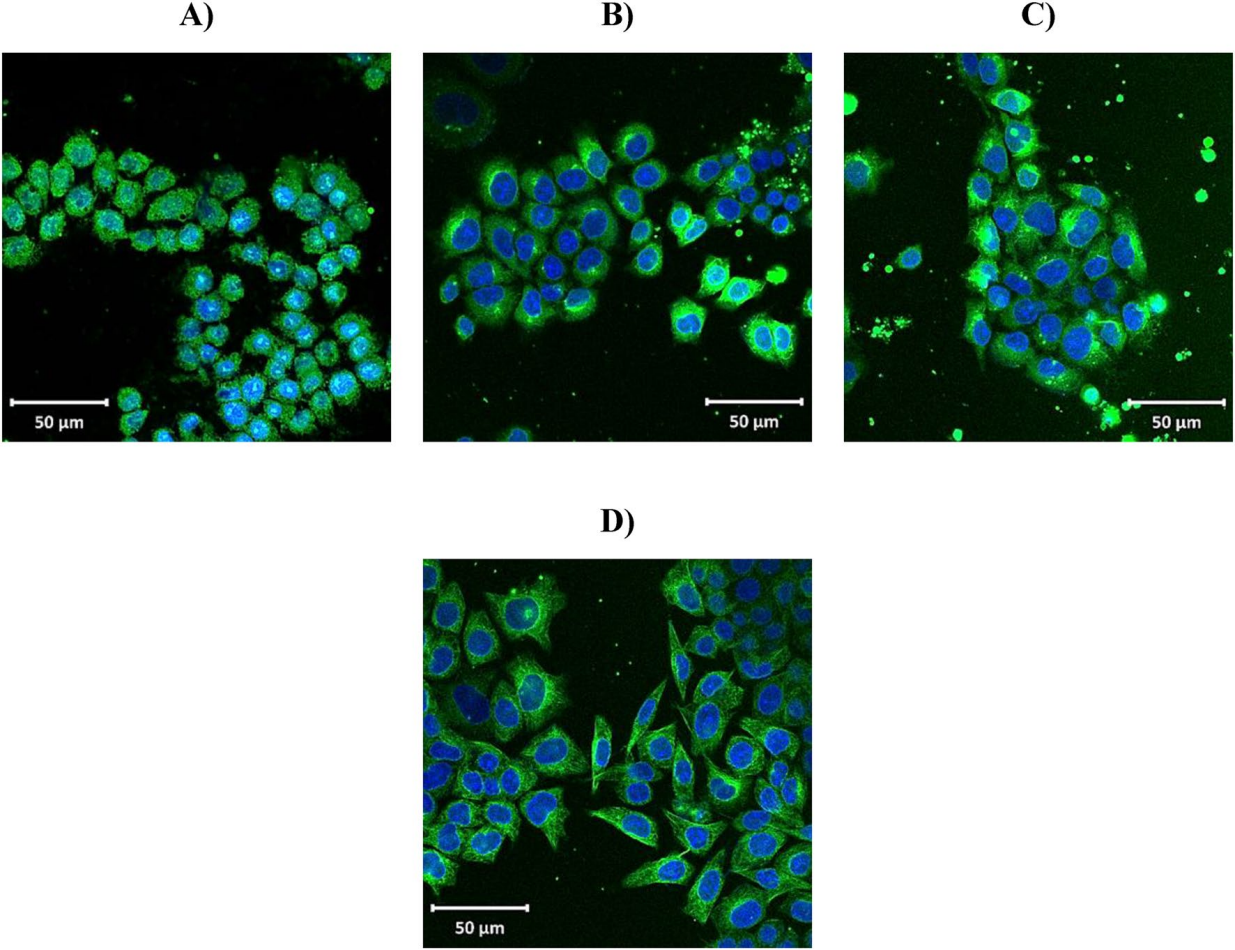
The effect of *Z. nummularia* ethanolic extract treatment on the tubulin network of KAIMRC2 cell line. Cells were treated with various doses of *Z. nummularia* ethanolic extract for 48 h and subsequently stained with Tubulin tracker™ Green (Cat # T34075) in HBSS for 30 min and HOECHST33342 for 5 min. *Z. nummularia* in ethanol extract 37.5 µg/ml (**A**). *Z. nummularia* in ethanol extract 75 µg/ml (**B**). *Z. nummularia* in ethanol extract 150 µg/ml (**C**). Mitoxantrone positive control (**D**). The images shown above are representative of three independent images.

**Figure 8:**
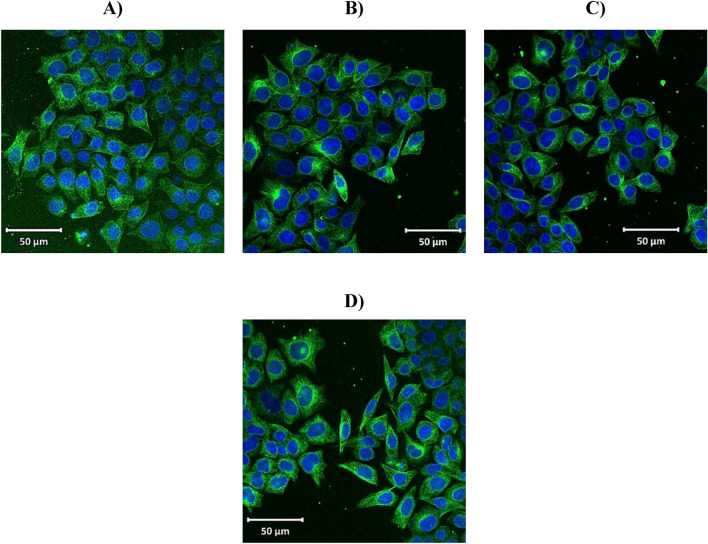
KAIMRC2 cell line tubulin network on treatment with *Z. nummularia* ethyl acetate extract. Cells were treated for 48 h and subsequently stained with Tubulin tracker™ Green (Cat # T34075) in HBSS for 30 min and HOECHST33342 for 5 min. *Z. nummularia* in ethyl acetate 37.5 µg/ml (**A**). *Z. nummularia* in ethyl acetate 75 µg/ml (**B**). *Z. nummularia* in ethyl acetate 150 µg/ml (**C**). Mitoxantrone positive control (**D**). The images shown above are representative of three independent images.

To examine the effect of *Z. nummularia* on microtubule network organization, the KAIMRC2 cell line was treated with three different concentrations of *Z. nummularia* ethanolic and ethyl acetate extracts. Mitoxantrone was used as the positive control. After treatment, the cells were stained with Tubulin Tracker™ Green (Cat # T34075). The ethanolic extract of *Z. nummularia* exhibited complete tubulin depolymerization, as shown in Fig. 7. However, the ethyl extract of *Z. nummularia* had little to no effect on the microtubular network structure as demonstrated in Fig. 8.

### Western blot

**Figure 9:**
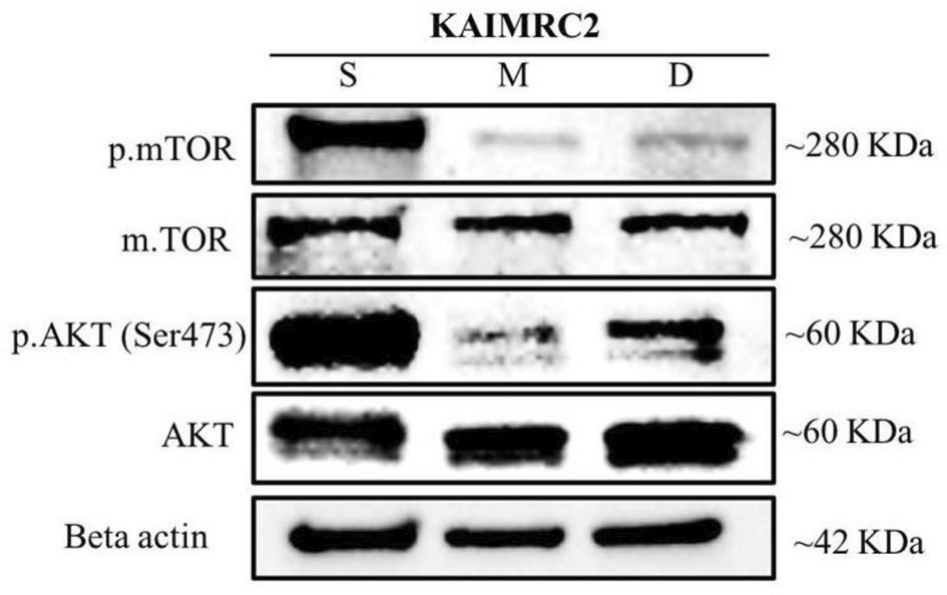
*Z. nummularia* ethanolic extracts induce mTOR and AKT phosphorylation. KAIMRC2 cells were treated with *Z. nummularia* ethanolic extract (**S**), Mitoxantrone (**M**), or DMSO (**D**) for 48 h. Cell lysates were analyzed by western blot for levels of indicated proteins. Beta-actin was used as a loading control. Results are representative of three independent experiments (Supplementary file 1).

To further investigate the cytotoxic mode of action of the ethanolic plant extract, KAIMRC2 cells were treated with *Z. nummularia* ethanolic extract, Mitoxantrone as a positive control, or DMSO as a negative control. Collected cell lysates were used to evaluate the activation of the mammalian target of rapamycin (mTOR) and the Akt serine/threonine kinase (AKT). Results show a significant upregulation of phosphorylated mTOR and AKT proteins in cells treated with the extract, as compared to the positive and negative controls, shown in Fig. 9**.**

### Identification of metabolites from *Z. nummularia* Ethanolic extract and ethyl acetate extract using QTOF-LCMS

**Figure 10:**
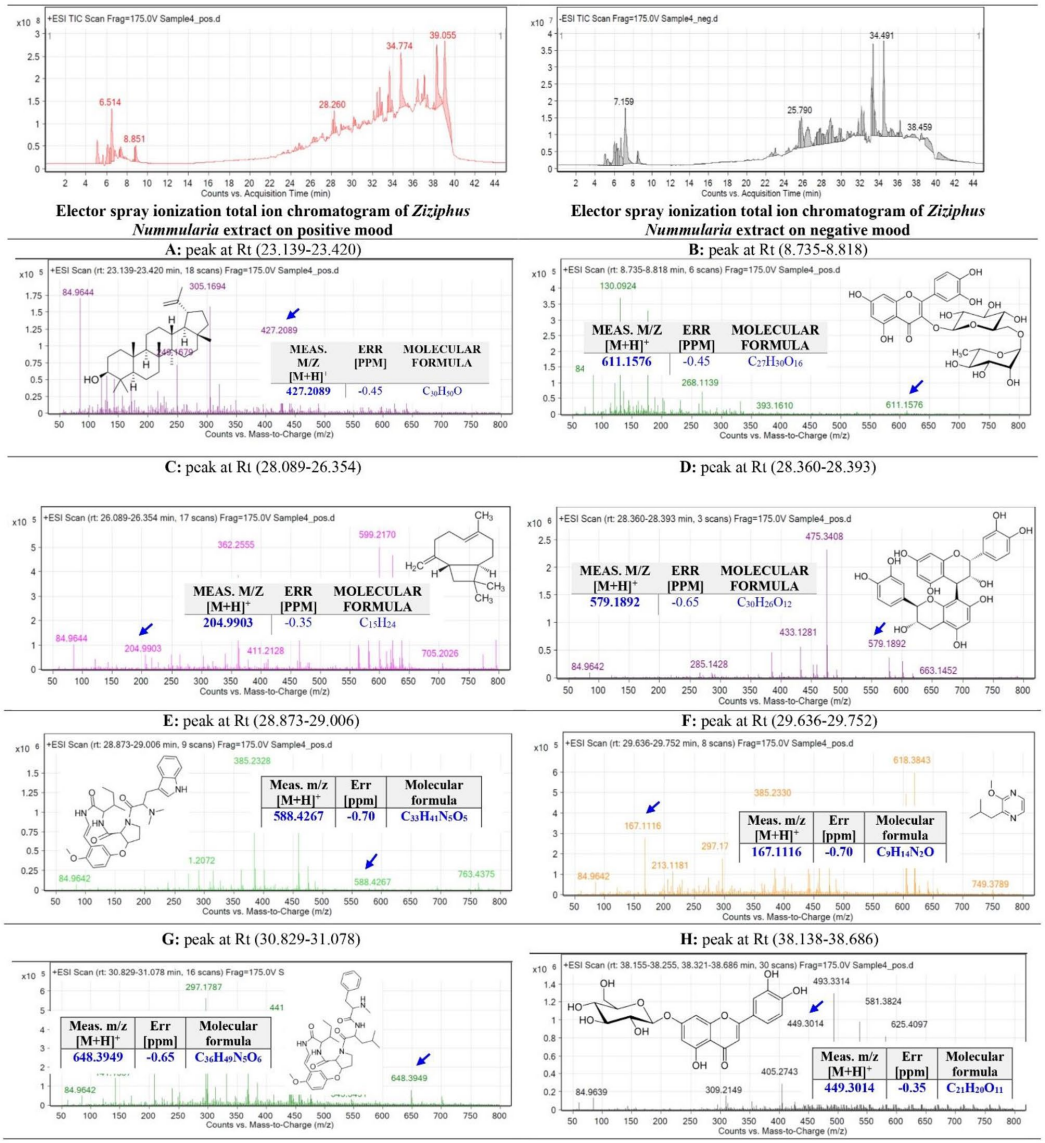
Base peak chromatogram of *Z. nummularia* ethanolic extract was extracted and tentatively identified secondary metabolites are (**A**) Lupeol^41^, (**B**) Rutin^42^, (**C**) Caryophyllene^43^ (**D**) Procyanidin B1^44^, (**E**) Nummularine R^45^,(**F**) 2-Isobutyl-3-methoxypyrazine^43^, (**G**) Nummularine A^46^, and (**H**) Luteolin-7-O-glucoside^44^ Means *m/z* implies measured.

**Figure 11:**
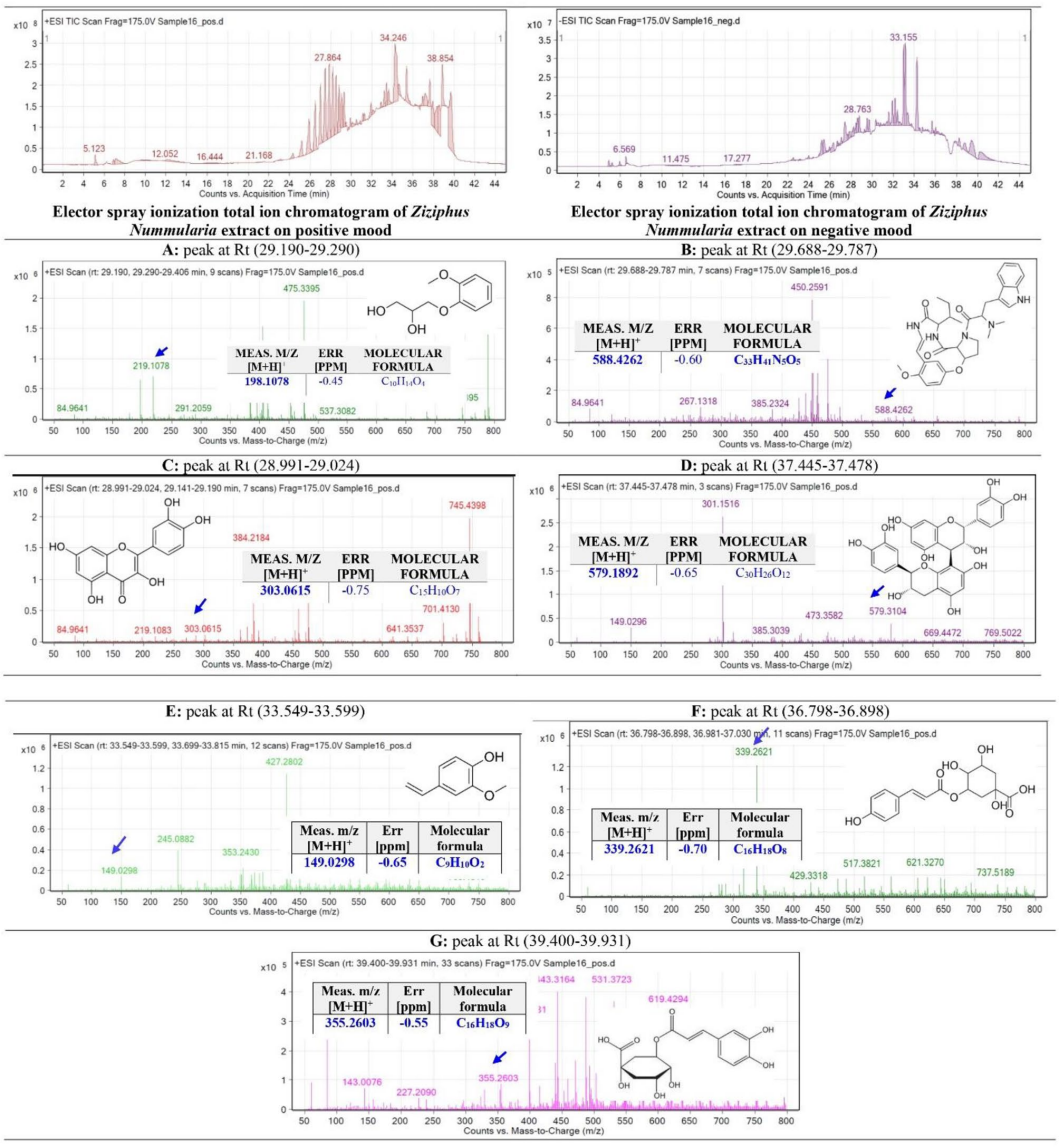
Base peak chromatogram of *Z. nummularia* ethyl acetate extract was tentatively identified secondary metabolites are (**A**) Guaifenesin^47^, (**B**) Nummularine R^45^, (**C**) Quercetin^41^, (**D**) Procyanidin B1^44^, (**E**) 2-Methoxy-4-vinylphenol^41^, (**F**) Coumaroylquinic acid^47^, and (**G**) Chlorogenic acid^41^, Means *m/z* implies measured *m/z*.

The ethanolic extract of *Z. nummularia* (Fig. 10) and the ethyl acetate extract of *Z. nummularia* (Fig. 11) were subjected to total ion current spectra (TIC). The data-analysis program Mass Hunter (Agilent Technologies) qualitative and quantitative analysis software was used for further analysis. After conducting a mass screening of the spectrums (Figs. 10 and 11) the chemical features were extracted from the LC–MS data using the Molecular Features Extraction (MFE) algorithm and the recursive analysis workflow. Features have been extracted by screening the detected nodes at various retention times per minute, with a minimum intensity of 6000, and counts and aligned with previously detected compounds considering adducts ([M + H]^+^, and [M − H]^−^).

The tentatively identified compounds from the ethanolic extract of *Z. nummularia* are Lupeol^41^, Rutin^42^, Caryophyllene^43^, Procyanidin B1^44^, Nummularine R^45^, 2-Isobutyl-3-methoxypyrazine^43^, Nummularine A^46^, and Luteolin-7-O-glucoside^44^ Means m/z implies measured m/z. The tentatively identified compounds from the ethyl acetate extract of *Z. nummularia* are Guaifenesin^47^, Nummularine R^45^, Quercetin^41^, Procyanidin B1^44^, 2-Methoxy-4-vinylphenol^41^, Coumaroylquinic acid^47^, and Chlorogenic acid^41^, Means *m/z* implies measured *m/z*.

### Prediction of activity spectra for substances (PASS)

The estimated biological activities of a compound can be predicted using the chemical structure of a molecule and computational tools such as the PASS online web server. The web server generates biological activity predictions using active-to-inactive ratios (*P*_a_: *P*_i_). Promising molecules are generally expected to exhibit values of putative activity (*P*_a_) greater than 0.5 (*P*_a_ > 0.5) and values of putative inactivity (*P*_i_) very close to zero. Table 4 shows the predicted biological activities of Z.* nummularia* metabolites identified from the plant's ethyl acetate and ethanolic extracts. While acknowledging that the metabolites Nummularine R and Procynadin B1 were present in both the ethanol and ethyl acetate extracts. We sought to include those metabolites in the results for the ethanol extract metabolites in the computational analysis to prevent repeating results.

**Table 4:** Predicted bioactivity scores for *Z. nummularia* metabolites in ethyl acetate and ethanol extracts.

Biological activities for metabolites	Anti-cancer activity	
	*P*_a_	*P*_i_
(Ethyl Acetate)		
Guaifenesin	0.263	0.066
Quercetin	0.797	0.012
2-Methoxy-4-Vinylphenol	0.617	0.042
3-P-Coumaroylquinic acid	0.779	0.014
Chlorogenic acid	0.778	0.014
(Ethanol)		
Lupeol	0.950	0.004
Rutin	0.983	0.001
Beta-Caryophyllene	0.915	0.005
Procyanidin B1	0.757	0.007
Nummularine R	0.390	0.117
2-Isobutyl-3-Methoxypyrazine	0.334	0.178
Nummularine A	0.416	0.093
Luteolin-7-O-Glucoside	0.935	0.002

Rutin, lupeol, beta-caryophyllene, and luteolin-7-O-glucoside showed significant *P*_a_ scores that were predicted to be greater than 90%, suggesting a potential biological anti-cancer activity for these metabolites.

### Prediction of the bioactivity score of identified metabolites in *Z. nummularia* ethanol and ethyl acetate extracts

The molecular targets for the metabolites identified from the ethyl acetate and ethanolic extracts of *Z. nummularia* were predicted using the online web server Molinspiration Chemoinformatics. The GPCR ligands, ion channel modulator, kinase inhibitor, nuclear receptor inhibitor, protease inhibitor, and other enzyme inhibitors were predicted as targets based on the prediction bioactivity scores shown in Table 5. A compound is likely to have significant biological activities if its bioactivity score is greater than 0.00, whereas values between − 0.50 and 0.00 are predicted to be moderately active, and less than − 0.50 is assumed to possess no activity.

**Table 5:** Molecular target predictions of identified metabolites in *Z. nummularia* ethyl acetate and ethanol extracts.

Molecular target	GPCR ligand	Ion channel modulator	Kinase inhibitor	Nuclear receptor inhibitor	Protease inhibitor	Enzyme inhibitor
Metabolites of ethyl acetate extract						
Guaifenesin	− 0.40	− 0.16	− 0.51	− 0.54	− 0.72	− 0.12
Quercetin	− 0.06	− 0.19	0.28	0.36	− 0.25	0.28
2-Methoxy-4-Vinylphenol	− 0.96	− 0.28	− 1.00	− 0.77	− 1.34	− 0.46
3-P-Coumaroylquinic acid	0.31	0.17	0.02	0.80	0.29	0.65
Chlorogenic acid	0.29	0.14	− 0.00	0.74	0.27	0.62
Metabolites of ethanol extract						
Lupeol	0.27	0.11	− 0.42	0.85	0.15	0.52
Rutin	− 0.05	− 0.52	− 0.14	− 0.23	− 0.07	0.12
Beta-Caryophyllene	− 0.34	0.28	− 0.78	0.13	− 0.60	0.19
Procyanidin B1	0.20	− 0.33	− 0.12	0.16	0.17	0.09
Nummularine R	0.22	− 0.46	− 0.17	− 0.45	0.44	− 0.09
2-Isobutyl-3-methoxypyrazine	− 0.39	− 0.34	− 0.51	− 0.98	− 1.01	− 0.33
Nummularine A	− 0.10	− 0.93	− 0.63	− 0.73	0.40	− 0.48
Luteolin-7-O-glucoside	0.09	− 0.02	0.15	0.27	− 0.01	0.42

The bioactivity score of 3-p-coumaroylquinic acid and chlorogenic acid indicated that both are GPCR ligands and/or ion channel modulators. The results further demonstrated that the kinase inhibitory activities of quercetin and 3-P-coumaroylquinic acid were significant. Chlorogenic acid, 3-P-coumaroylquinic acid, and quercetin demonstrated significant predictions of nuclear receptor inhibition. Only 3-P-coumaroylquinic acid and chlorogenic acid exhibited protease-inhibitory properties. Finally, an enzyme inhibitory activity was predicted for quercetin, 3-p-coumaroylquinic acid, and chlorogenic acid.

The data in Table 5 suggest that lupeol, procyanidin B1, Nummularine R, and luteolin-7-O-glucoside are GPCR ligand modulators. The only metabolites that were predicted to modulate ion channels were lupeol and beta-caryophyllene. luteolin-7-O-glucoside was the only compound predicted to inhibit kinase receptors. lupeol, beta-caryophyllene, procyanidin B1, and luteolin-7-O-glucoside all were predicted to target and inhibit nuclear receptors. lupeol, procyanidin B1, Nummularine R, and Nummularine A were predicted to be protease inhibitors. Finally, lupeol, rutin, beta-caryophyllene, procyanidin B1, and luteolin-7-O-Glucoside were predicted to have enzyme inhibitory activity.

### Molecular docking analysis of the metabolites identified for *Z. nummularia* ethanol extract

**Figure 12:**
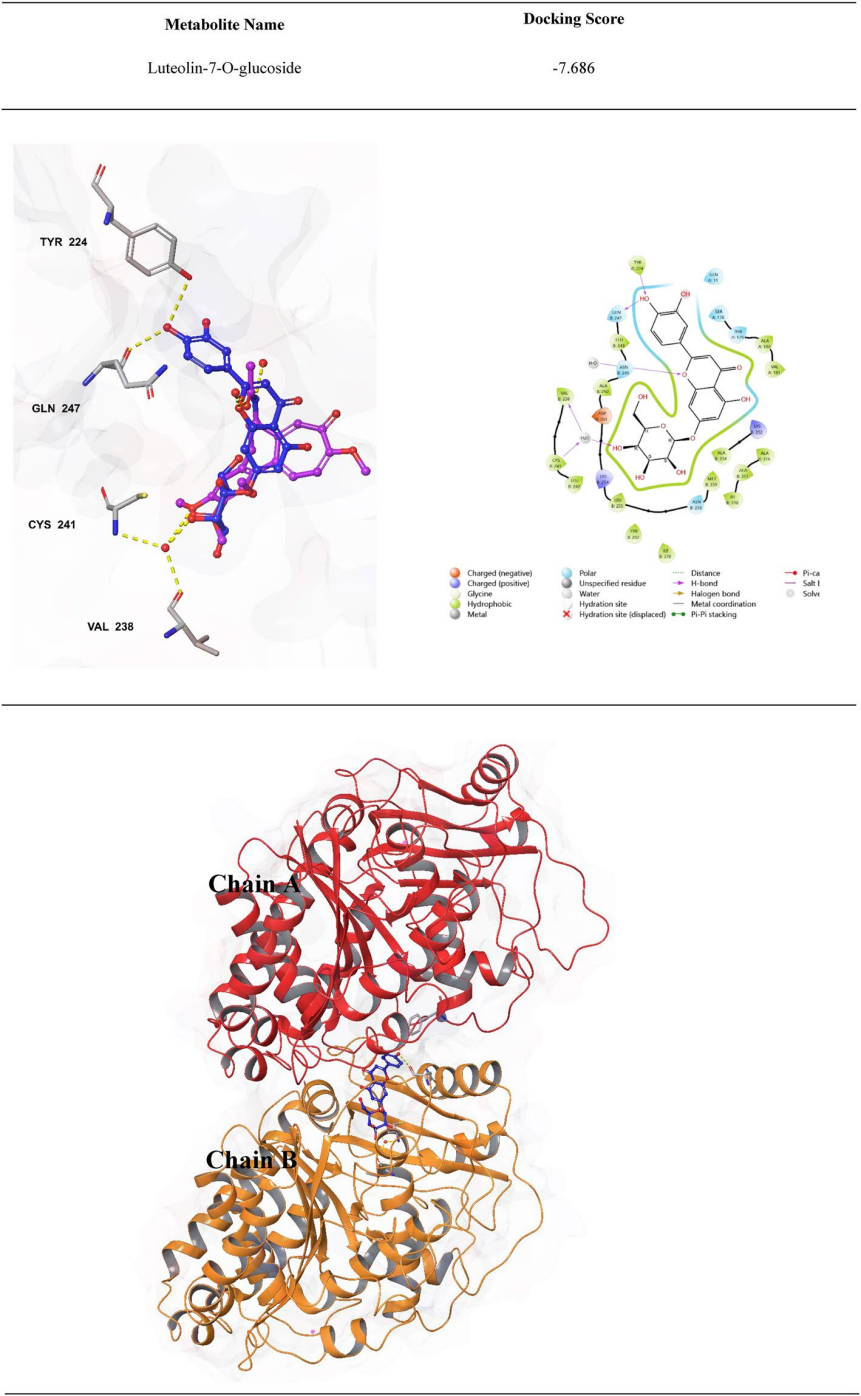
2D and 3D binding interactions of luteolin-7-O-glucoside against Tubulin binding site. The hydrogen bond interactions are indicated by the yellow dashed lines. The color of luteolin-7-O-glucoside is blue, while colchicine is violet.

From our experimental findings, it was apparent that *Z. nummularia* extract showed remarkable inhibition of tubulin polymerization. Thus, we investigated the molecular basis of this inhibition and assessed the important interactions at the binding site. To achieve this goal, molecular docking was conducted for the metabolites identified in *Z. nummularia* extracts with the tubulin crystal structure. All the metabolites were docked, and luteolin-7-O-glucoside demonstrated the best docking score (-7.686) and interactions relative to the others. The docking scores for the remaining metabolites ranged from -7.1 to -3.2. Luteolin-7-O-glucoside formed four Hydrogen Bond (H-bond) interactions with TYR 224, VAL 238, CYS 241, and GLN 247 in the binding site, as shown in Fig. 12. Moreover, luteolin-7-O-glucoside occupied a binding site comparable to that of the native ligand.

#### Pharmacokinetic parameters evaluation of secondary metabolites in *Z. nummularia* ethyl acetate and ethanol extracts

The SwissADME webserver was utilized as a tool to evaluate the pharmacokinetic parameters of the ethyl acetate and ethanol extracts of* Z. nummularia*. According to Lipinski's role in drug-likeness (the possibility that a chemical will be orally active in terms of bioavailability is measured by its drug-likeness), there is a greater risk of poor absorption when the molecular weight (MW) is greater than 500, there are 10 H-bond acceptors (HBA), and more than five H-bond donors (HBD). Physicochemical properties such as molecular weight between 150 and 500 g/mol, solubility (log S not higher than 6), lipophilicity (XLOGP3 between − 0.7 and + 5.0), flexibility (less than 9 rotatable bonds), and saturation (fraction of carbons in the sp3 hybridization not less than 0.25) predicted that the molecule was orally active. The pharmacokinetic parameters of the *Z. Nummularia* ethyl acetate extract metabolites, as shown in Table 6, revealed that all molecular weights fell within the range of 150 and 500 g/mol. Guaifenesin and 2-Methoxy-4-vinylphenol displayed an HBA of less than 5, with all metabolites having less than 10 HBD bonds. Additionally, the XLOGP3 values ranged from -0.7 to + 5.0 for all metabolites, indicating their lipophilicity. Guaifenesin, quercetin, and 2-Methoxy-4-vinylphenol exhibited significant gastrointestinal absorption, while 2-Methoxy-4-vinylphenol was the only metabolite permeable through the blood–brain barrier (BBB). Moreover, quercetin inhibited CYP1A2, CYP2D6, and CYP3A4, whereas 2-Methoxy-4-vinylphenol inhibited only CYP1A2. The only molecule that violated the Lipinski rule of five (NHorOH > 5) was chlorogenic acid.

**Table 6:** Pharmacokinetic properties of the identified metabolites from *Z. nummularia* in ethyl acetate extract using the SwissADME webserver.

Properties	Parameters	Guaifenesin	Quercetin	2-Methoxy-4-vinylphenol	3-P-coumaroylquinic acid	Chlorogenic acid
Physicochemical properties	MW (G/Mol)	198.22	302.24	150.17	338.31	354.31
	HBA	4	7	2	8	9
	HBD	2	5	1	5	6
Lipophilicity Log P_o*/W*_	iLOGP	1.60	1.63	2.14	0.88	0.96
	XLOGP3	1.39	1.54	2.81	− 0.07	− 0.42
	MLOGP	0.37	− 0.56	1.71	− 0.54	− 1.05
Absorption	Water Solubility	− 1.98	− 3.24	− 2.38	− 0.18	0.40
		(Soluble)	(Soluble)	(Soluble)	(Soluble)	(Soluble)
	GI	High	High	High	Low	Low
	Log Kp (Skin Permeation) Cm/S	− 6.52	− 7.05	− 5.22	− 8.41	− 8.76
Distribution	BBB Permeant	No	No	Yes	No	No
Metabolism	CYP1A2 Inhibitor	No	Yes	Yes	No	No
	CYP2C19 Inhibitor	No	No	No	No	No
	CYP2C9 Inhibitor	No	No	No	No	No
	CYP2D6 Inhibitor	No	Yes	No	No	No
	CYP3A4 Inhibitor	No	Yes	No	No	No
Druglikeness	Lipinski	Yes; 0 Violation	Yes; 0 Violation	Yes; 0 Violation	Yes; 0 Violation	Yes; 1 Violation: NHorOH > 5

Table 7 shows the pharmacokinetic parameters of the metabolites from the *Z. nummularia* ethanol extract. Lupeol, beta-caryophyllene, 2-isobutyl-3-methoxypyrazine, and luteolin-7-O-glucoside all had molecular weights within the range (150 and 500 g/mol). Lupeol, beta-caryophyllene, and 2-isobutyl-3-methoxypyrazine HBA were less than 5. Rutin and procyanidin B1 HBD was 10, but the remaining metabolites HBD were less than 10. Only lupeol showed an XLOGP3 value out of the recommended range (-0.7 to + 5.0). In addition, lupeol, Nummularine R, and Nummularine A showed poor water solubility. While rutin (− 0.29), beta-caryophyllene (− 3.77), procyanidin B (− 3.91), 2-isobutyl-3-methoxypyrazine (− 3.00), and luteolin-7-O-glucoside (− 2.10) demonstrate water solubility. In terms of GI absorption, Nummularine R, 2-isobutyl-3-methoxypyrazine, and Nummularine A seem to have high GI absorption. 2-isobutyl-3-methoxypyrazine seems to cross the BBB. Regarding the CYP enzyme inhibition, CYP1A2 is likely to be inhibited by 2-isobutyl-3-methoxypyrazine. Beta-caryophyllene showed inhibition of CYP2C19 and CYP2C9. While CYP3A4 was inhibited by procyanidin B, Nummularine R, and Nummularine A. 2-isobutyl-3-methoxypyrazine was the only metabolite with zero violations to the Lipinski role of five.

**Table 7:** Pharmacokinetic properties for the identified metabolites of *Z. nummularia* in ethanol using SwissADME Webserver.

Properties	Parameters	Lupeol	Rutin	Beta-Caryophyllene	Procyanidin B1	Nummularine R	2-Isobutyl-3-methoxypyrazine	Nummularine A	Luteolin-7-O-glucoside
Physicochemical Properties	MW (g/mol)	426.72	610.52	204.35	578.52	587.71	166.22	647.80	448.38
	HBA	1	16	0	12	6	3	7	11
	HBD	1	10	0	10	3	0	4	7
Lipophilicity Log Po/w	iLOGP	4.68	1.58	3.28	2.05	3.74	2.32	4.34	1.83
	XLOGP3	9.87	− 0.33	4.38	2.37	4.33	2.59	4.80	1.46
	MLOGP	6.92	− 3.89	4.63	− 0.26	1.16	0.66	1.15	− 2.10
Absorption	Water solubility	− 6.74	− 0.29	− 3.77	− 3.91	− 7.68	− 3.00	− 8.31	− 2.10
		(Poorly soluble)	(Soluble)	(Soluble)	(Soluble)	(Poorly soluble)	(Soluble)	(Poorly soluble)	(Soluble)
	GI	Low	Low	Low	Low	High	High	High	Low
	Log Kp (skin permeation) cm/s	− 1.90	− 10.26	− 4.44	− 8.15	− 6.81	− 5.48	− 6.84	− 8.00
Distribution	BBB Permeant	No	No	No	No	No	Yes	No	No
Metabolism	CYP1A2 inhibitor	No	No	No	No	No	Yes	No	No
	CYP2C19 inhibitor	No	No	Yes	No	No	No	No	No
	CYP2C9 inhibitor	No	No	Yes	No	No	No	No	No
	CYP2D6 inhibitor	No	No	No	No	No	No	No	No
	CYP3A4 inhibitor	No	No	No	Yes	Yes	No	Yes	No
Druglikeness	Lipinski	Yes; 1 violation: MLOGP > 4.15	No; 3 violations: MW > 500, NorO > 10, NHorOH > 5	Yes; 1 violation: MLOGP > 4.15	No; 3 violations: MW > 500, NorO > 10, NHorOH > 5	Yes; 1 violation: MW > 500	Yes; 0 violation	No; 2 violations: MW > 500, NorO > 10	No; 2 violations: NorO > 10, NHorOH > 5

The biological activities of metabolites at CYP isoenzymes (substrate/non substrate) were examined (Table 8), revealing their interactions with specific enzymes. Guaifenesin, Quercetin, 2-Methoxy-4-Vinylphenol, and several others were identified as substrates for CYP1A2, CYP2C19, CYP2C9, CYP2D6, and CYP3A4. Conversely, compounds such as 3-P-Coumaroylquinic Acid, Chlorogenic acid, Lupeol, and others were classified as non-substrates for various CYP isoenzymes. These findings provide valuable insights into the metabolic pathways and potential drug interactions involving these metabolites.

**Table 8:** The biological activities of metabolites at CYP isoenzymes as substrate or non-substrate.

Biological activities for metabolites at CYP isoenzymes	CYP isoenzymes				
	CYP1A2	CYP2C19	CYP2C9	CYP2D6	CYP3A4
(Ethyl Acetate)					
Guaifenesin	Substrate	Substrate	Substrate	Substrate	Substrate
Quercetin	Substrate	Substrate	Substrate	Substrate	Substrate
2-Methoxy-4-Vinylphenol	Substrate	Substrate	Substrate	Substrate	Substrate
3-P-Coumaroylquinic Acid	Non-substrate	Non-substrate	Substrate	Substrate	Substrate
Chlorogenic acid	Non-substrate	Non-substrate	Substrate	Substrate	Substrate
(Ethanol)					
Lupeol	Non-substrate	Substrate	Substrate	Non-substrate	Substrate
Rutin	Substrate	Non-substrate	Substrate	Non-substrate	Substrate
Beta-Caryophyllene	Non-substrate	Substrate	Substrate	Non-substrate	Substrate
Procyanidin B1	Substrate	Substrate	Substrate	Substrate	Substrate
Nummularine R	Non-substrate	Non-substrate	Non-substrate	Non-substrate	Non-substrate
2-Isobutyl-3-Methoxypyrazine	Non-substrate	Non-substrate	Non-substrate	Non-substrate	Non-substrate
Nummularine A	Non-substrate	Non-substrate	Non-substrate	Non-substrate	Non-substrate
Luteolin-7-O-Glucoside	Substrate	Non-substrate	Substrate	Non-substrate	Substrate

**Figure 13:**
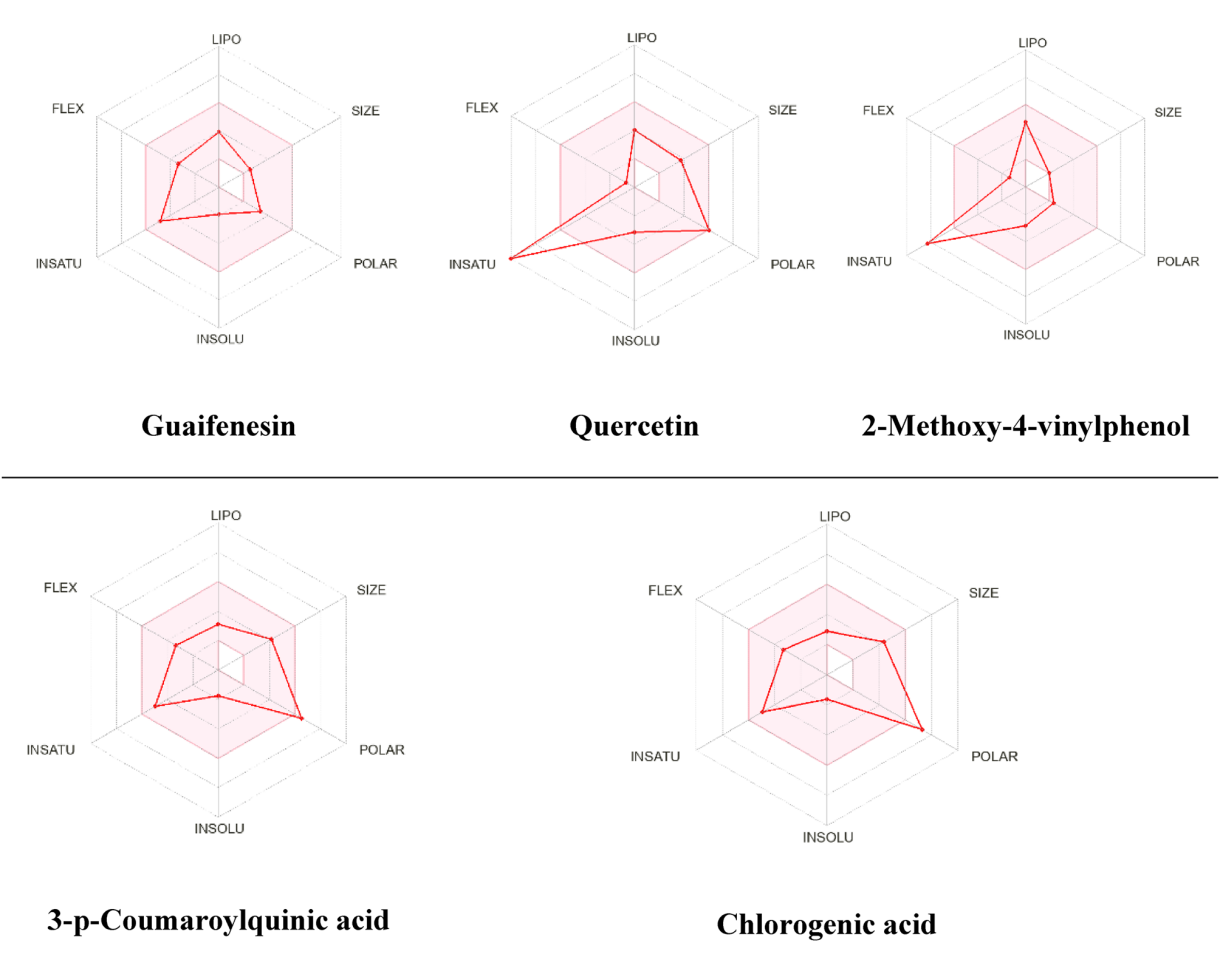
Bioavailability radar charts of secondary metabolites identified in *Z. nummularia* ethyl acetate extract.

**Figure 14:**
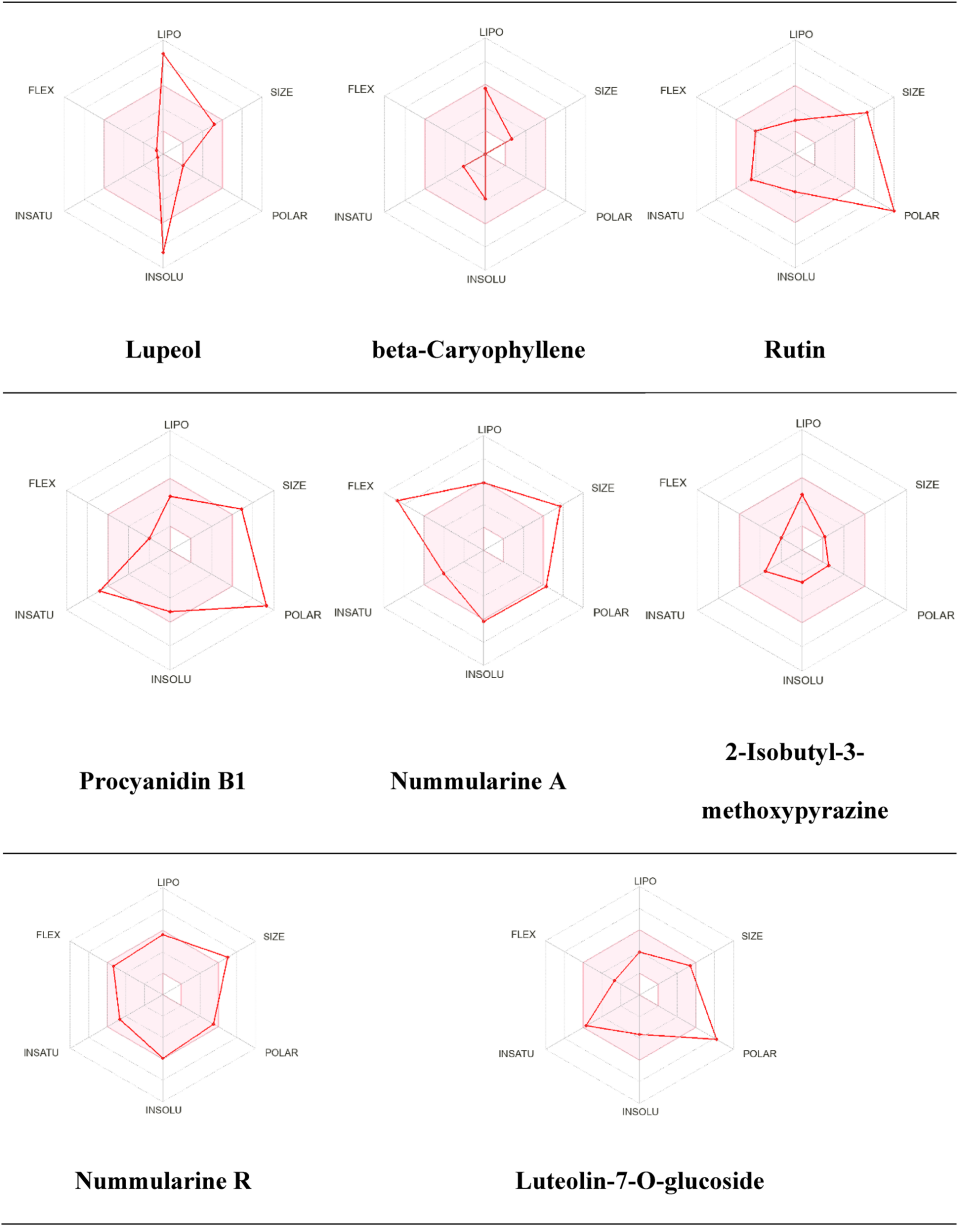
Bioavailability radar charts of secondary metabolites identified in *Z. nummularia* ethanol extract.

In addition, the oral bioavailability was assessed and evaluated for the identified metabolites using the bioavailability radar. The parameters included polarity (POLAR), solubility (INSOLU), lipophilicity (LIPO), flexibility (FLEX), saturation (INSATU), and size (SIZE). As illustrated in Fig. 13, the red line represents the predicted properties for the evaluated molecules identified in the *Z. nummularia* ethyl acetate extract, while the pink-shaded region represents the recommended range for the properties to be orally active. Guaifenesin is predicted to be orally bioavailable. Oral bioavailability was predicted to be low for quercetin, 2-Methoxy-4-vinylphenol, 3-p-Coumaroylquinic acid, and chlorogenic acid. Figure 14 shows the bioavailability radar for *Z. nummularia* ethanol extract. Beta-caryophyllene and 2-isobutyl-3-methoxypyrazine oral bioavailability was predicted to be within the recommended range for oral bioavailable.

#### Toxicity evaluation of secondary metabolites in *Z. nummularia* ethyl acetate and ethanol extracts

In the drug discovery process, it is important to evaluate toxicological profiles before reaching the clinical stage. Thus, the identified metabolites were subjected to computational assessment and evaluation of their organ and endpoint toxicities, including hepatotoxicity, cytotoxicity, carcinogenicity, mutagenicity, and immunotoxicity.

As summarized in Table 9, all metabolites of *Z. nummularia* ethyl acetate were predicted to be inactive as hepatotoxic or cytotoxic agents. However, only quercetin showed carcinogenicity and mutagenicity. All the metabolites except quercetin and guaifenesin were predicted to be immunotoxic. The ethanol extract of *Z. nummularia* did not appear to be hepatotoxic, carcinogenic, mutagenic, or cytotoxic. Only lupeol, rutin, beta-caryophyllene, procyanidin B1, and Nummularine R were predicted to have immunotoxic activity.

**Table 9:** Toxicity Evaluation of the identified metabolites in *Z. nummularia* ethyl acetate and ethanol extracts using the Protox II Web Server.

Metabolite name	Organ toxicity (%Probability)	Toxicity endpoint (% Probability)			
	Hepatotoxicity	Carcinogenicity	Immunotoxicity	Mutagenicity	Cytotoxicity
Guaifenesin	Inactive	Inactive	Inactive	Inactive	Inactive
	(0.99)	(0.81)	(0.86)	(0.73)	(0.90)
Quercetin	Inactive	Active	Inactive	Active	Inactive
	(0.69)	(0.68)	(0.87)	(0.51)	(0.99)
2-Methoxy-4-Vinylphenol	Inactive	Inactive	Active	Inactive	Inactive
	(0.66)	(0.61)	(0.59)	(0.97)	(0.88)
3-P-Coumaroylquinic Acid	Inactive	Inactive	Active	Inactive	Inactive
	(0.75)	(0.70)	(0.97)	(0.93)	(0.80)
Chlorogenic Acid	Inactive	Inactive	Active	Inactive	Inactive
	(0.72)	(0.68)	(0.99)	(0.93)	(0.80)
Lupeol	Inactive	Inactive	Active	Inactive	Inactive
	(0.91)	(0.63)	(0.57)	(0.95)	(0.97)
Rutin	Inactive	Inactive	Active	Inactive	Inactive
	(0.80)	(0.91)	(0.98)	(0.88)	(0.64)
Beta-Caryophyllene	Inactive	Inactive	Active	Inactive	Inactive
	(0.80)	(0.70)	(0.54)	(0.95)	(0.75)
Procyanidin B1	Inactive	Inactive	Active	Inactive	Inactive
	(0.74)	(0.54)	(0.92)	(0.59)	(0.80)
Nummularine R	Inactive	Inactive	Active	Inactive	Inactive
	(0.61)	(0.55)	(0.99)	(0.66)	(0.52)
2-Isobutyl-3-Methoxypyrazine	Inactive	Inactive	Inactive	Inactive	Inactive
	(0.64)	(0.60)	(0.98)	(0.75)	(0.84)
Nummularine A	Inactive	Inactive	Inactive	Inactive	Inactive
	(0.63)	(0.61)	(0.73)	(0.64)	(0.54)
Luteolin-7-O-glucoside	Inactive	Inactive	Inactive	Inactive	Inactive
	(0.82)	(0.85)	(0.74)	(0.76)	(0.69)

#### Prediction of cardiac toxicity

The pred-hERG webserver was used to predict the cardiac toxicity of the metabolites. Green atoms on the map indicate contributions to hERG blockage, pink indicates that it assists in reducing hERG blockage, and gray indicates the location of a split between positive (green) and negative (pink) contributions. As summarized in Table 10, our results demonstrate that all identified ethyl acetate metabolites exhibit no cardiac toxicity, as evidenced by the confidence (50–60% range) and probability map. Pred-hERG predicted that three out of five compounds were nontoxic with an accuracy of 60%, and two out of five compounds were nontoxic with an accuracy of 50%. However, secondary metabolites from the *Z. nummularia* ethanolic extract showed potential cardiotoxicity ( +). Lupeol, beta-caryophyllene, procyanidin B, Nummularine A, and luteolin-7-O-glucoside had a 50% confidence level. Rutin, Nummularine R, and 2-isobutyl-3-methoxypyrazine showed a 60% confidence level.

**Table 10:** Cardiac toxicity of the identified metabolites using Pred-hERG web server.

Metabolite number	Prediction/potency	Confidence (%)	Probability map
Guaifenesin	Non-cardiotoxic (−)	60	
Quercetin	Non-cardiotoxic (−)	60	
2-Methoxy-4-Vinylphenol	Non-cardiotoxic (−)	60	
3-p-Coumaroylquinic acid	Non-cardiotoxic (−)	50	
Chlorogenic acid	Non-cardiotoxic (−)	50	
Lupeol	Potential cardiotoxic (+)	5	
Rutin	Potential cardiotoxic (+)	60	
beta-Caryophyllene	Potential cardiotoxic (+)	50	
Procyanidin B1	Potential cardiotoxic (+)	50	
Nummularine R	Potential cardiotoxic (+)	60	
2-Isobutyl-3-methoxypyrazine	Potential cardiotoxic (+)	60	
Nummularine A	Potential cardiotoxic (+)	50	
Luteolin-7-O-glucoside	Potential cardiotoxic (+)	50	

#### Endocrine distruptome predictions

The endocrine disruption profile of* Z. nummularia* metabolites was evaluated. Nuclear receptor binding was used by the Endocrine Disruptome (open-source prediction tool) to assess the potential for endocrine disruption. The results in Table 11 show the predicted binding of the identified metabolites to different receptors. Red indicates a high likelihood of binding, orange and yellow suggest a medium likelihood of binding, and green indicates a low likelihood of binding. According to the data in Table 11 for ethyl acetate and ethanolic extracts of *Z. nummularia*. Quercetin was the only compound with a high probability of androgen receptor binding and antagonism. On the other hand, lupeol, and rutin were identified with glucocorticoid receptor antagonism activity.

**Table 11:**
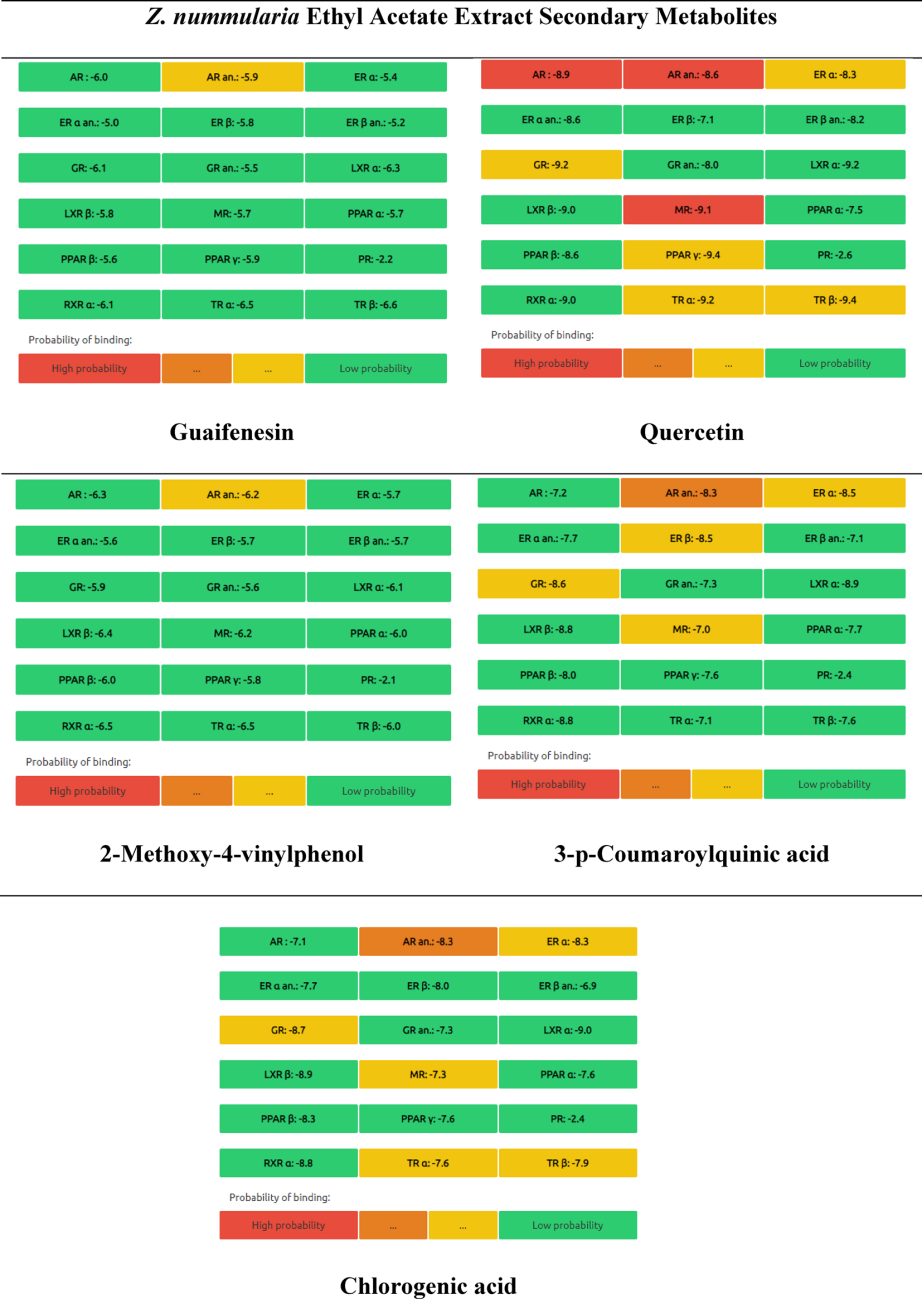

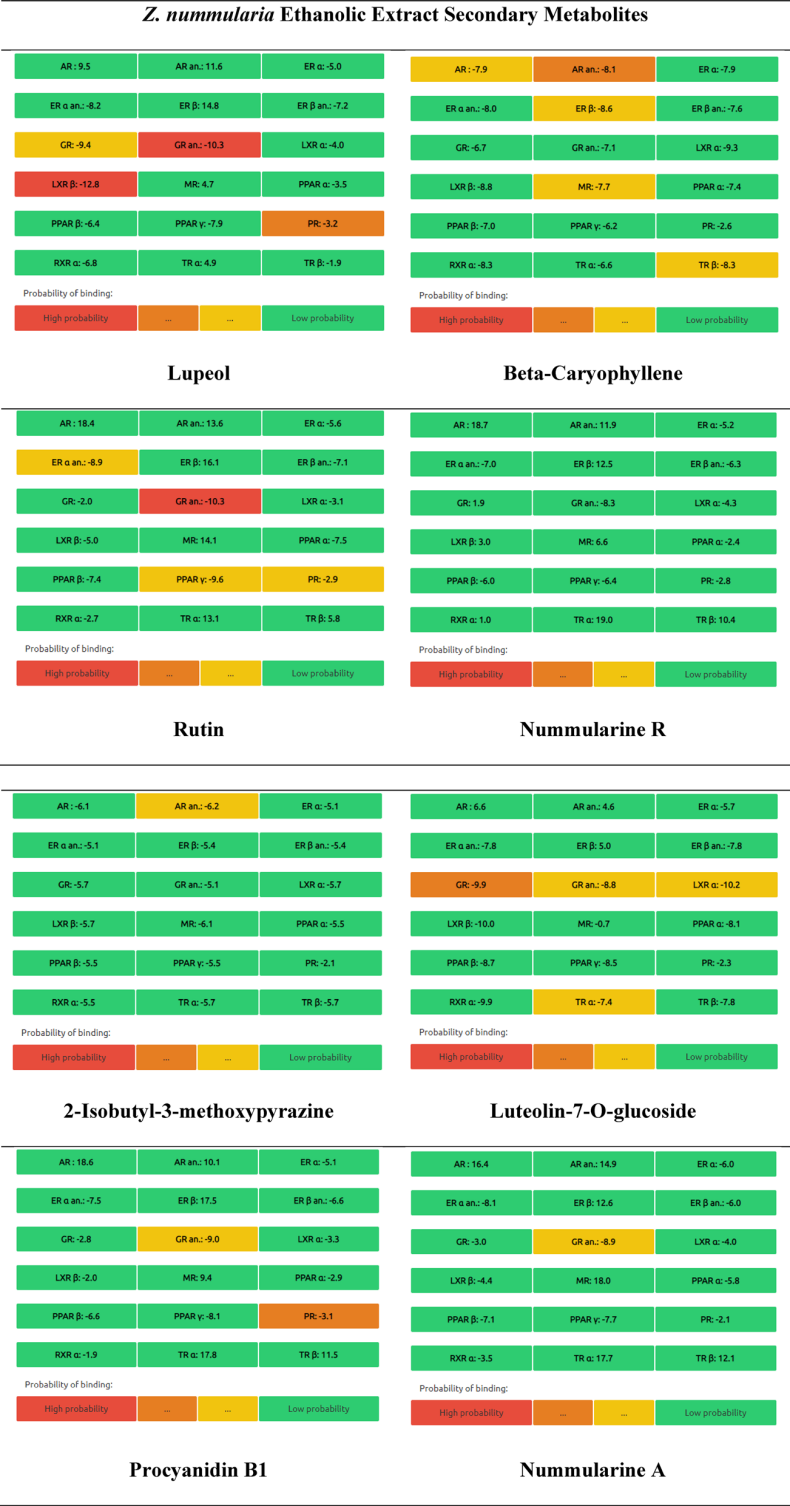
Endocrine distruptome predictions of the identified metabolites in *Z. nummularia* ethanol and ethyl acetate extract.

## Discussion

### In vitro biological activities of *Z. nummularia* and its metabolites

Drug entities can be discovered using natural or synthetic variants with novel structures. For instance, 40 of the 75 small molecules studied between 1946 and 1980 are natural products used in the field of cancer treatment, and many medications used for cancer treatment are plant-derived, such as vincristine and paclitaxel^48,49^. Therefore, medicinal plants are considered a valuable source/reservoir for bioactive compounds. The extraction process is considered a crucial step in obtaining bioactive phytochemicals compounds. The polarity of the extraction solvent affects the solubility and biological activity of a compound. Previous studies have discovered a significant relationship between the solvent used and the biological effects of extracts (e.g., antioxidants)^50^, and a recent in vitro study showed that total phenolic and anthocyanin contents varied depending on the solvent^50,51^.

In general, water, methanol, ethanol, and acetone are used to extract hydrophilic polyphenols (e.g., glycosides). However, alcoholic solutions are optimal for successful polyphenolic extractions^52^. Polyphenols are more stable in relatively lower pH solvents due to their neutrality in acidic environments^52–54^. Consequently, the current study showed that ethanolic extracts have the most potent cytotoxicity effects on cancer cells, whereas aqueous extracts of the plants proved to have little or no cytotoxic effect. This could be due to the findings that phenolic compounds (e.g., lupeol, rutin, and luteolin-7-O-glucosides) are more abundant in ethanolic extracts than in aqueous extracts. Here we show that the choice between ethyl acetate and ethanol as an extraction solvent dictates the metabolites present in the extract. The only two metabolites common to both the ethanol and ethyl acetate extracts were Procyanidin B1 and Nummularine R. Each solvent could extract unique metabolites having distinctive biological properties. For instance, the ethyl acetate extract contained Guaiphenesin, which was not detected in the ethanol extract. Guaiphenesin is well-known and widely used as a cough expectorant. Our studies show that the ethanolic extract was the most cytotoxic, whereas the other extracts had far fewer cytotoxic effects. Lupeol, which was found in the ethanolic extract, has various pharmacological properties, including anticancer and antimicrobial properties^55^. Lupeol promoted NK cell proliferation and inhibited gastric cancer cell lines BGC823 and N87, presumably by an enhanced mechanism^55,56^. Rutin was also identified in Z.* nummularia* ethanolic extract. Rutin has been shown to promote angiogenesis and stimulate cell cycle arrest and apoptosis^57,58^. The DPPH and FRAP scavenging assay were used to evaluate the radical-scavenging activity of β-caryophyllene, and the results demonstrated the antioxidant efficacy of β-caryophyllene^59^. Procyanidin B1 was shown to reduce the levels of NF-κB protein and possesses an anti-inflammatory effect^60–62^.

The current study also shows the wide range of anti-cancer activities. Our ApoTox-Glo™ Triplex Assay findings showed that the ethanolic and ethyl acetate extracts of *Z. nummularia* induce a dose-dependent cytotoxic effect on KAIMRC2 breast cancer and HCT8 colon cancer cells. In addition, it was visually observable (HCI-apoptosis assay) that KAIMRC2 cells and HCT8 cells were undergoing apoptosis on treatment with increasing doses of the ethanolic extract of *Z. nummularia*. However, in our case in the ApoTox-Glo™ Triplex Assay, we did not see any significant effect of inducing apoptosis; although our positive control also did not show a positive apoptosis result. Since the ApoTox-Glo Triplex Assay is a time-dependent assay we believe that the period for observing the caspase 3 activity in this assay preceded the time of detection. Previous studies also point to the sub-optimal detection time to be a cause of an apparent negative apoptotic response^63^. Indeed, luteolin is reported to induce apoptosis via caspase activation, activating caspase-8, caspase-9, and caspase-3 in a dose-dependent manner^64^.

The ethanolic extract of *Z. nummularia* significantly upregulated phosphorylated Akt and mTOR proteins in KAIMRC2 breast cancer cells, thus switching on the AKT/mTOR pathway, which is traditionally known to promote cell survival. Previous studies highlight the ability of luteolin-7-O-glucoside, a metabolite found in the ethanolic extract of *Z. nummularia,* also to activate the AKT pathway in nasopharyngeal carcinoma cell lines^65^. Although Akt activation is related to cellular survival, it has been shown that hyperactivation of AKT can promote apoptosis^66^. mTOR regulates autophagy and can induce HIF-1α expression, which promotes survival and may consequently lead to a more resistive and aggressive cancer phenotype^24,67^. Since the current study shows that treatment with *Z. nummularia* ethanolic extract upregulates AKT/ mTOR activity, it suggests an adaptive response to treatment to allow repair and promote cancer cell survival. Hyperactivation of mTOR is known to be related to chemoresistance, particularly cisplatin resistance^68^ and abated clinical therapeutic effects are traditionally known to be a problem associated with using chemotherapeutic agents. Although there are well-known mechanisms of cancer adaptation or resistance, such as drug inactivation, alteration of the drug target, drug efflux, autophagy, reduced susceptibility to apoptosis, altered proliferation, and epithelia-mesenchymal transition^69^. A highly conceivable point to consider in all these processes is time; where a stronger, more efficacious agent, which provides a sharp, robust response, may avoid the tangible effects of allowing the cancer cells time to adapt to the drug environment. Very little is known about the time aspect of pharmacological therapeutics, which is dictated by the pharmacodynamics and pharmacokinetics within the cell. One modality off-set resistive mechanisms could be to combine the use of herbal agents with an mTOR inhibitor, which can lead to more efficacious therapeutic effects^70^. Furthermore, the activity of AKT and mTOR is known to promote necroptosis^26,71^, which may be considered as an alternative hypothesis to explain the activation of AKT/mTOR in the KAIMRC2 cells upon treatment with the herbal extract, which correlates with the induction of cytotoxicity. Further research assessing ROS levels, markers for phenotypic changes, and autophagy, in addition to other key characteristics of the cell, are necessary to fully understand and evaluate the response of the KAIMRC2 on treatment with *Z. nummularia* ethanolic extract.

Luteolin has been reported to inhibit insulin-stimulated phosphorylation of the insulin receptor-beta subunit (IR-beta) and insulin-stimulated Akt activation^72^. Insulin stimulates protein synthesis and cell growth via Akt activation, and other insulin-like growth factors are known to promote angiogenesis, cellar proliferation, and differentiation^73,74^. It may be that luteolin and the *Z. nummularia* extracts effectively modulate and initiate apoptosis in insulin and insulin-like growth factor-dependent tumors. Data from a previous study identified that insulin receptors are highly expressed in KAIMRC2 cells^75^. Therefore another important area of study would be to investigate the effects of herbal-derived active metabolites in insulin and insulin-like growth factor-dependent cancers.

Moreover, primary work demonstrated that KAIMRC2 exhibited significant upregulation of E-cadherin^75^. E-cadherin can activate the intracellular PI3K signaling pathway, providing a different pathway to promote actin (part of the cytoskeleton structure essential for cell mobility and cell division) polymerization^76^. The current data suggest that the ethanolic extract of *Z. nummularia* promotes cytotoxicity by destabilizing and dismantling the tubulin network in the KAIMRC2 cell lines, which could disrupt communication, cytoskeletal changes damaging the structure and integrity of the cells, as well as preventing mitosis during cell division. Various drugs such as taxanes and vinca alkaloids target tubulin polymerization, and suppressing tubulin polymerization seems to be a fundamental method for developing novel, effective anticancer medications^77^. Although further studies assessing the cytotoxic activity of the ethanolic extract of *Z. nummularia* on a diverse panel and phenotypically distinguished cancer cells are necessary, the active metabolites may be a viable option for further research and development to treat many cancers.

### In silico predictions of biological activities, molecular targets, safety profiles and pharmacokinetic assessment of *Z. nummularia* metabolites

In silico interpretations of biological activity spectrums (BAS) assist in the early stages of research by excluding biomolecules with potentially negative pharmacological properties. The PASS assessment demonstrated a promising anti-cancer activity for the identified metabolites from the ethyl acetate Z.* nummularia* extract, particularly with chlorogenic acid, which exhibited the highest predicted anti-cancer activity. Our results were consistent with previous studies that concluded that chlorogenic acid had strong anti-cancer activities, causing reduced cancer cell migration, invasion, growth, and ATP synthesis in mitochondria^78^. A similar prediction was obtained with 2-methoxy-4-vinylphenol indicating the potential cytotoxic activity of *Z. nummularia* metabolites*.* Moreover, according to the Molinspiration web server, 3-p-coumaroylquinic acid and chlorogenic acid were predicted to be nuclear receptor ligands, suggesting the potentiality that 3-p-coumaroylquinic acid and chlorogenic acid could also be investigated as new anti-cancer therapy against steroid-dependent tumors, and leukemias^79^.

The molecular docking study was performed to assess and visualize the molecular interactions between *Z. nummularia* metabolites and tubulin crystal structure. The predicted binding mode and interaction of luteolin-7-O-glucoside with the tubulin binding site was interesting due to the high similarity in binding mode and interactions with the native ligand (colchicine). These results provide additional confirmation for the observed inhibition of tubulin polymerization via *Z. nummularia* metabolites which could account for its anti-cancer activity.

An effective drug must reach its target in the body sufficiently for it to be used as a medication. Early ADME parameter prediction in the discovery phase may significantly lower the percentage of pharmacokinetics-related failure in the clinical phases. Hence the ADME profile of *Z. nummularia* metabolites was investigated^80^. All the metabolites from *Z. nummularia* ethyl acetate extract demonstrated no permeability to penetrate the BBB except 2-methoxy-4-vinylphenol. Metabolites from the ethanol extract also exhibited no BBB penetration except for 2-isobutyl-3-methoxypyrazine. This finding indicates that most of the investigated metabolites lack any negative side effects on the CNS and are predicted to act peripherally.

Regarding the metabolites from the ethyl acetate extract, guaifenesin, quercetin, and 2-methoxy-4-vinylphenol all possessed a high ratio of GIT absorption and most of the metabolites did not violate Lipinski's rule of five (ROF), which suggests that those metabolites may be effective bioavailable oral medications. As mentioned earlier, Guaiphenesin is consumed orally and clinically as an expectorant. In the case of the metabolites from the ethanolic extract, the ROF values were mostly violated. The gastrointestinal (GI) absorption predictions were poor for all the metabolites, including luteolin-7-O-glucoside, except for Nummularine R, 2-isobutyl-3-methoxypyrazine, and Nummularine A. Therefore, structural modifications may be necessary for most of the metabolites from the *Z. nummularia* ethanolic extract to improve the predicted values for oral administration and bioavailability.

Additionally, drug metabolism often requires the involvement of CYP enzymes, and it’s through the CYP450 enzymes that many drug-drug interactions occur. Inhibition or activation of these enzymes could potentially result in dangerous health consequences. Our findings suggest that the compounds guaifenesin, 3-p-coumaroylquinic acid, and chlorogenic acid do not inhibit CYP1A2, CYP2C19, CYP2C9, CYP2D6, and CYP3A4 and hence these compounds are predicted to produce fewer CYP mediated drug-drug interactions. Moreover, the toxicity profile screening indicated that most of the metabolites lack toxicity except for 2-methoxy-4-vinyl phenol, 3-p-coumaroylquinic acid, and chlorogenic acid, which were predicted to be immunotoxins. The secondary metabolites from the ethanolic extract did not inhibit the CYP enzyme, including luteolin-7-O-glucoside, suggesting a relatively safe application for patients on polypharmacy.

The investigation into the biological activities of metabolites at different CYP isoenzymes has yielded significant insights. The substrates identified for CYP1A2, CYP2C19, CYP2C9, CYP2D6, and CYP3A4 include Guaifenesin, Quercetin, 2-Methoxy-4-Vinylphenol, among others. Conversely, compounds such as 3-P-Coumaroylquinic Acid, Chlorogenic acid, Lupeol, and others were found to be non-substrates for various CYP isoenzymes.

These findings have implications for drug metabolism and potential drug interactions. Understanding the interactions between metabolites and CYP isoenzymes is crucial for predicting the metabolic fate of drugs and for designing effective and safe pharmaceutical interventions. Further research in this area could lead to the development of improved drug metabolism prediction models and the identification of potential drug-drug interactions, ultimately contributing to the advancement of personalized medicine and drug safety.

Before a biomolecule can be utilized as a drug candidate, the Food and Drug Administration (FDA) mandates that it be evaluated for human Ether-à-go-go-Related Gene (hERG) safety. Drug-induced blockage of hERG channels can be regarded as an important cause of cardiotoxicity. Researchers have recently become interested in hERG due to its association with QT interval elongation, leading to ventricular arrhythmia (torsades de pointes or TdP), ventricular fibrillation, and sudden death^81^. All of the ethyl acetate metabolites were predicted to possess no negative effects on the heart and no effect on the hERG K^+^ channels. On the other hand, metabolites from the *Z. nummularia* ethanolic extract may exert potential cardiac adverse effects, necessitating further chemical modifications of the structure to improve its safety while monitoring its efficacy.

The biological activity of hormones may be mimicked by chemical compounds, and this resemblance could result in unwanted side effects related to the modulation of endocrine receptors. Most of the ethyl acetate extract metabolites appear to have little effect on the endocrine hormone receptors except quercetin, which was predicted to have a high probability of being an antagonist at the androgen receptor. This finding suggests that quercetin might be used as an AR inhibitor^82^. Previous research suggests that some patients with androgen-driven TNBC may benefit from an AR-targeted treatment^82^. In addition, lupeol and rutin from the ethanolic extract of* Z. nummularia* were predicted to act as antagonists at the glucocorticoid receptor, which implies that lupeol and rutin could be used as GR inhibitors. Previous studies have found that an important mediator of aggressive TNBC progression is phospho-GR. Authors suggested that GR can drive TNBC migration, invasion, anchorage-independent cell growth, and tumor sphere formation^83^. Furthermore, GR may function similarly in other endocrine-related and/or highly metastatic forms of neoplasia since GR is a widely distributed steroid hormone receptor present in various malignancies such as prostate cancer, ovarian cancer, and melanoma^83^.

## Conclusion

*Z. nummularia* is a herb containing an array of secondary metabolites that can be differentially extracted using various solvents. Ethanol is an optimal solvent for solubilizing metabolites having anticancer activity. Luteolin-7-O-glucoside is the most active anti-cancer metabolite, having anti-tubulin activity and binding properties similar to the native tubulin ligand. As such, Luteolin-7-O-glucoside may be a lead metabolite for further development and structure–activity-based research to increase its safety profile and efficacy. Furthermore, our research suggests the ability of cancer cells to switch on adaptive mechanisms of survival and growth on treatment with *Z. nummularia* extract, which poses a question regarding the unlabeled use of herbs in the treatment of cancers and its effects on cancer growth and resistance.

### Supplementary Information

Supplementary Information.

## Data Availability

The data, information of metabolites and collected plant samples presented in this study are available on request from the corresponding author. The plants samples were collected after obtained KAIMRC permission. The plants identifications were confirmed by a botanist and the samples were deposited at King Saud University (Herbium. Department of Botany and Microbiology) with accession numbers 24176 and 24,322. All data generated during this study are presented in the article.

## Abbreviations

ADME: Absorption, distribution, metabolism, and excretion
AR: Androgen receptor
BAK: Bcl-2 homologous antagonist/killer
BAS: Biological activity spectrums
BAX: Bcl-2-associated X protein
BBB: Blood-brain barrier
BCL-2: B-cell lymphoma protein 2
BCL-XL: B-cell lymphoma-extra large
BSA: Bovine serum albumin
CNS: Central nervous system
CO_2_: Carbon dioxide
CYP1A2: Cytochrome P450 family 1 subfamily A member 2
CYP3A4: Cytochrome P450 family 3 subfamily A member 4
CYP2D6: Cytochrome P450 family 2 subfamily D member 6
DISC: Death-inducing signaling complex
DMEM: Dulbecco’s modified eagle medium
DMSO: Dimethyl sulfoxide
DoTS: Docking interface for the target system
DPPH: 1,1-Diphenyl-2-picrylhydrazyl (DPPH)-2,2-diphenyl-1-picrylhydrazyl
ER: Estrogen receptor
FBS: Fetal bovine serum
FLEX: Flexibility
FRAP: Ferric reduction activity potential
GI: Gastrointestinal
GPCR: G protein-coupled receptor
GR: Glucocorticoid receptor
HBA: H-bond acceptors
HBD: H-bond donors
H-bond: Hydrogen bond
hERG K^+^: Human ether-a-go-go-related gene
INSATU: Saturation
INSOLU: Solubility
IR-beta: Insulin receptor-beta subunit
LC–MS: Liquid chromatography–mass spectrometry
LIPO: Lipophilicity
LXR: Liver X receptors
MAPs: Microtubule-associated proteins
MCL-1: Myeloid cell leukemia-1
MFE: Molecular features extraction
MR: Mineralocorticoid receptor
mTOR: Mammalian target of rapamycin
mTORC1: Mammalian target of rapamycin complex 1
mTORC2: Mammalian target of rapamycin complex 2
MTT reagent: 3-(4,5-Dimethylthiazol-2-yl)-2,5-diphenyl-2H-tetrazolium bromide
MW: Molecular weight
NAD(P)H: Nicotinamide adenine dinucleotide phosphate
NF-κB: Nuclear factor kappa B
NK: Natural killer
PASS: Prediction of activity spectra for substance
PBS: Phosphate-buffered saline
PDB: Protein data bank
PI3K: Phosphatidylinositol-3 kinase
PKB: Protein kinase B
POLAR: Polarity
PPAR: Peroxisome proliferator-activated receptors
PR: Progesterone receptor
PVDF: Polyvinylidene fluoride
ROF: Lipinski's rule of five
RXR: Retinoid X receptor
SIZE: Size
TIC: Total ion current
TNBC: Triple-negative breast cancer
TNF: Tumor necrosis factor
TR: Thyroid receptors
*Z. nummularia*: *Ziziphus nummularia*
*Z. spina-christi*: *Ziziphus spina-christi*
