# Supplementary material for: Exploring the potential of Ziziphus nummularia and luteolin-7-O-glucoside as tubulin inhibitors in cancer therapy and survival
Source: Sci Rep. 2024 Mar 26;14:7202. doi: 10.1038/s41598-024-57680-0 (PMC10966015; doi:10.1038/s41598-024-57680-0)

Western blot raw images

mTOR and p.mTOR

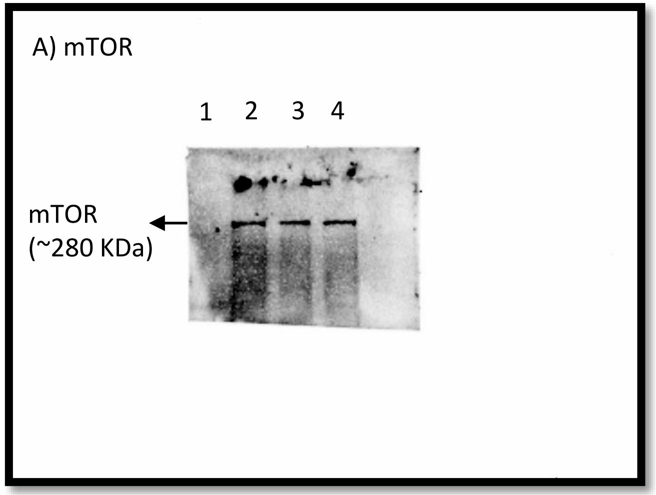

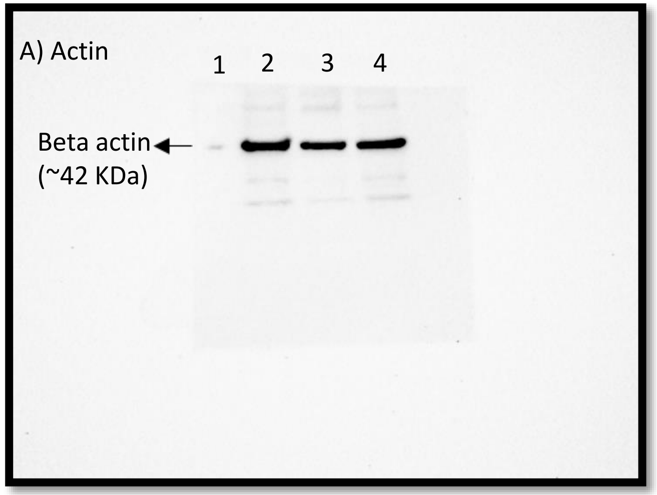

Panel A: Western blot images for KAIMRC2 cells showing the expression of our protein of interest (mTOR) and the house keeping gene (Beta actin).
Lane1: Precision Plus protein standard
Lane 2: KAIMRC2 cell lysate treated with *Z. nummularia* ethanolic extract
Lane 3: KAIMRC2 cell lysate with Mitoxantrone
Lane 4: KAIMRC2 cell lysate with DMSO

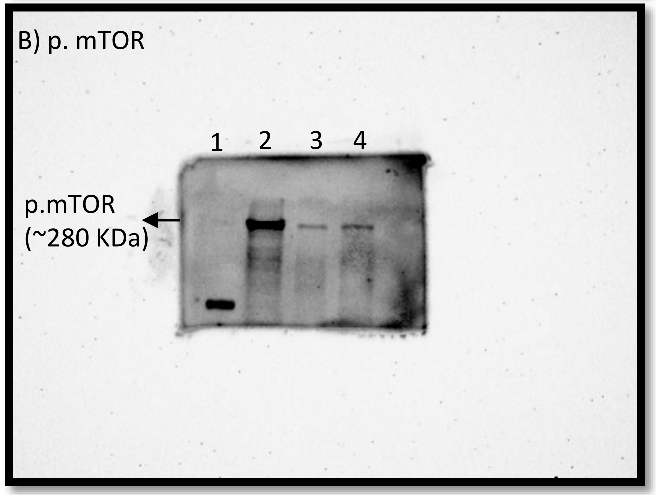

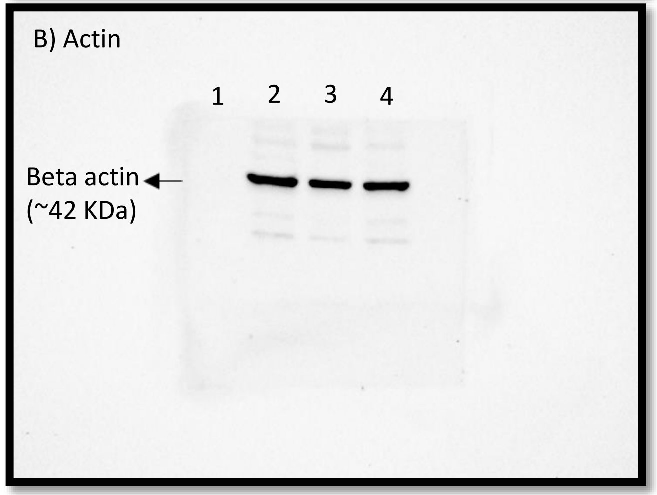

Panel B: Western blot images for KAIMRC2 cells showing the expression of our protein of interest (p. mTOR) and the house keeping gene (Beta actin).
Lane1: Precision Plus protein standard
Lane 2: KAIMRC2 cell lysate treated with *Z. nummularia* ethanolic extract
Lane 3: KAIMRC2 cell lysate with Mitoxantrone
Lane 4: KAIMRC2 cell lysate with DMSO

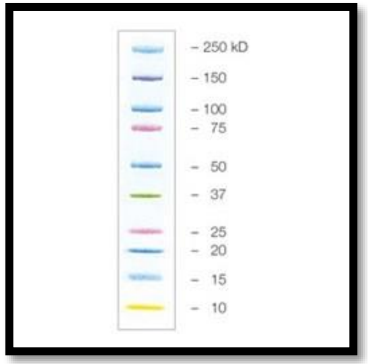

AKT and p. AKT (Ser437)

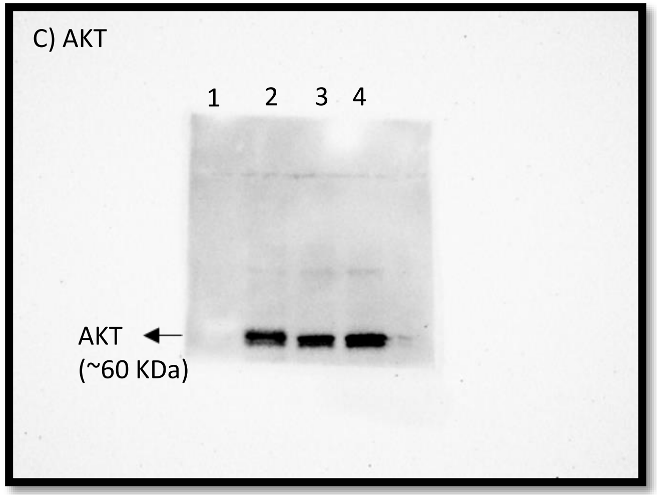

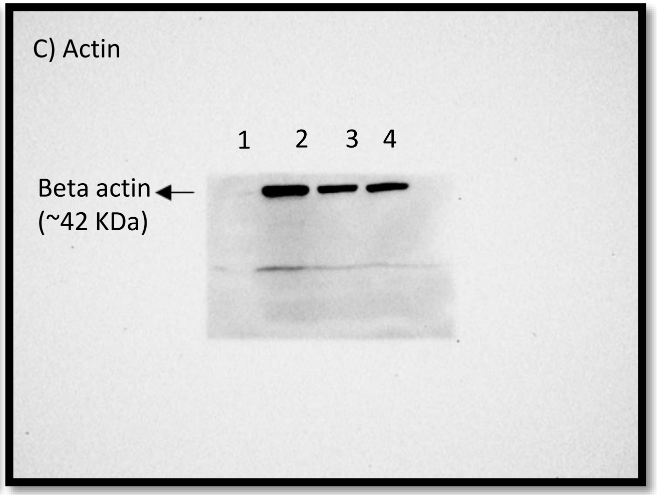

Panel C: Western blot images for KAIMRC2 cells showing the expression of our protein of interest (AKT) and the house keeping gene (Beta actin).
Lane1: Precision Plus protein standard
Lane 2: KAIMRC2 cell lysate treated with *Z. nummularia* ethanolic extract
Lane 3: KAIMRC2 cell lysate with Mitoxantrone
Lane 4: KAIMRC2 cell lysate with DMSO

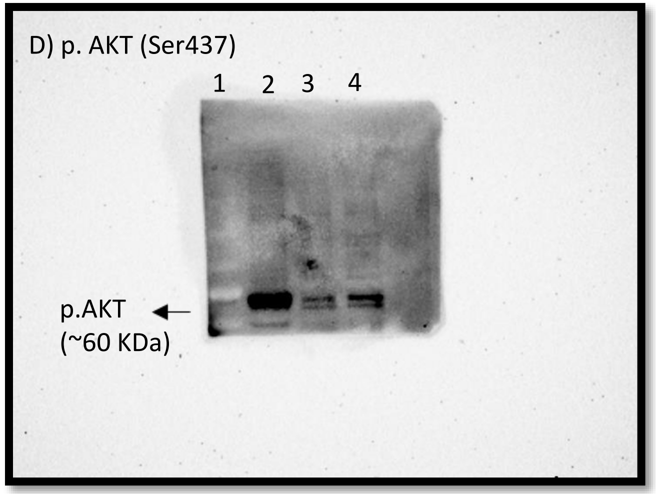

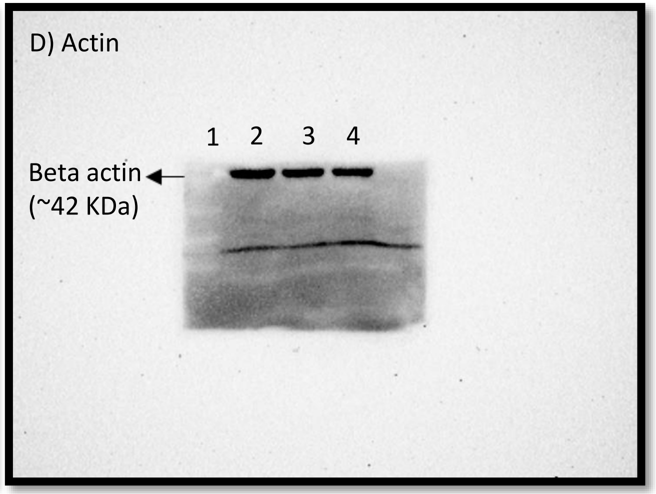

Panel D: Western blot images for KAIMRC2 cells showing the expression of our protein of interest (p. AKT) and the house keeping gene (Beta actin).
Lane1: Precision Plus protein standard
Lane 2: KAIMRC2 cell lysate treated with *Z. nummularia* ethanolic extract
Lane 3: KAIMRC2 cell lysate with Mitoxantrone
Lane 4: KAIMRC2 cell lysate with DMSO

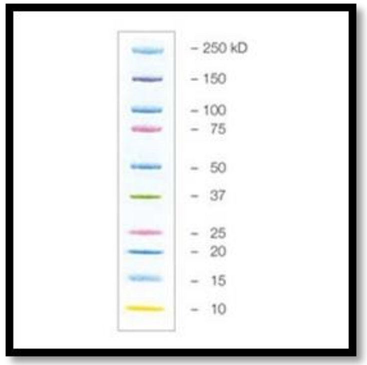

Supplement: Supplementary file 1 — Supplementary Information. [file 41598_2024_57680_MOESM1_ESM.pdf]
